# Microbiome Adaptation Could Amplify Modeled Projections of Global Soil Carbon Loss With Climate Warming

**DOI:** 10.1111/gcb.70301

**Published:** 2025-06-19

**Authors:** Elsa Abs, Scott R. Saleska, Steven D. Allison, Philippe Ciais, Yang Song, Michael N. Weintraub, Regis Ferriere

**Affiliations:** ^1^ Department of Ecology and Evolutionary Biology University of Arizona Tucson Arizona USA; ^2^ Department of Ecology and Evolutionary Biology University of California Irvine California USA; ^3^ Laboratoire Des Sciences du Climat et de L'environnement, LSCE/IPSL, CEA‐CNRS‐UVSQ Université Paris‐Saclay Gif‐sur‐Yvette France; ^4^ Department of Earth System Science University of California Irvine California USA; ^5^ Department of Hydrology and Atmospheric Sciences University of Arizona Tucson Arizona USA; ^6^ Department of Environmental Sciences University of Toledo Toledo Ohio USA; ^7^ International Center for Interdisciplinary Global Environmental Studies (IGLOBES), CNRS, Ecole Normale Supérieure, Paris Sciences & Lettres University University of Arizona Tucson Arizona USA; ^8^ Institut de Biologie de L'ENS (IBENS), CNRS, INSERM, Ecole Normale Supérieure Paris Sciences & Lettres University Paris France

**Keywords:** climate carbon feedback, eco‐evolutionary processes, global soil carbon, global warming, microbiome adaptation, soil carbon decomposition

## Abstract

Warming alters soil microbial traits through ecological and evolutionary processes, directly influencing the decomposition of organic matter, which significantly affects global soil carbon emissions. Yet, soil carbon models largely ignore these processes and their implications for global responses to warming. Here, we incorporate eco‐evolutionary theory into a mechanistic model describing microbial soil carbon decomposition to address the question of whether such processes could have consequential effects on climate carbon feedbacks globally. We assume that a key trait of microbes, their resource allocation to production of exoenzymes (which facilitate decomposition of organic matter)—is optimized to environmental temperatures by natural selection. We find that eco‐evolutionary optimization results in microbes allocating more resources to enzyme production under warming. When applied at the global scale, eco‐evolutionary optimization enhances the biological realism of soil carbon models and significantly amplifies global soil carbon loss by 2100. Our results highlight the significant potential of microbial eco‐evolutionary responses to influence carbon cycle feedbacks to climate change, and motivate an urgent need for more comprehensive data to accurately quantify the adaptive potential of microbiomes in response to climate change.

## Graphical abstract

Microbes in soil play a key role in breaking down organic matter and releasing carbon into the atmosphere. This study shows that as the climate warms, microbes may adapt by producing more enzymes that speed up decomposition, potentially doubling global soil carbon loss by 2100. Including microbial adaptation in climate models could significantly change our predictions of future carbon emissions from soils.

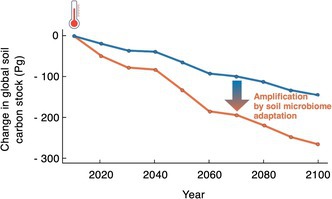

## 1 Introduction

Microorganisms are key drivers of global biogeochemistry, including the conversion of terrestrial soil organic matter into atmospheric CO_2_ through decomposition (Crowther et al. 2019). Experimental and observational studies indicate that a warming‐induced increase in microbial activity has the potential to increase soil carbon losses and amplify climate‐warming (Blankinship et al. 2011; Castro et al. 2010; Melillo et al. 2017; Walker et al. 2018). Though some global earth system models (ESMs) now include microbial physiology and functional diversity across space and time (Shevliakova et al. 2024), they still omit eco‐evolutionary dynamics, the ability of microbial communities to adjust to changing environmental conditions through environmental selection of variants more suited to the new conditions (Abs, Chase, et al. 2024; Abs et al. 2023; Abs and Ferrière 2020; Chase et al. 2021; Glassman et al. 2018; Malik et al. 2020).

Microbial eco‐evolutionary dynamics refer to the variation over time in the physiological and functional traits of microbial communities, as shaped by a combination of ecological shifts (taxa selection, dispersal), evolutionary processes (mutation, horizontal gene transfer, natural selection, random drift) and their interactions (Abs, Albright, and Allison 2024; Nemergut et al. 2013). Evolutionary game theory and its extensions to general ecological scenarios (Doebeli and Dieckmann 2000; Metz et al. 1992) provide a framework to predict how functional traits may respond to environmental change (Defrenne et al. 2021). This approach predicts the trait response as the outcome of an optimization process, i.e., maximizing the relative fitness of populations as environmental conditions change. A previous application to terrestrial carbon cycling (Weng et al. 2017, 2019) represented eco‐evolutionary processes in plants via a dynamic vegetation model that optimized leaf phenological strategies in response to changing soil nutrient resources to predict carbon flows from plants to the atmosphere.

We focus our analysis on a key microbial trait driving soil organic matter decomposition: the allocation of carbon resources by microbes to the production of extracellular enzymes (exoenzymes). Exoenzymes diffuse locally in the soil from the microbes that produce them, breaking down soil organic matter compounds that are too large for direct microbial uptake (Ratledge 1994). Empirical data support the existence of genetically based variation in exoenzyme production ([Guo et al. 2020; Trivedi et al. 2016] and see Supporting Information S1). Producing more exoenzymes makes new resources available to the community, but with the potential cost paid by producers who allocate carbon to exoenzyme production instead of their own growth (Harder and Dijkhuizen 1983). At the community level, fitness benefits (larger resource pool) may accrue to both producers and nonproducers that do not incur a growth cost for producing enzymes (Velicer 2003). We thus expect variation in exoenzyme production capacity to show strong responses to selection pressures (Guo et al. 2020; Rainey and Rainey 2003; Trivedi et al. 2016; Velicer 2003).

In this study, we address the following two questions: Can microbial eco‐evolution significantly alter global soil carbon projections over the next century in response to climate warming? If so, is this effect spatially homogeneous, or does it vary nonlinearly with temperature across regions? To answer these questions, we incorporate microbial eco‐evolutionary adaptation into a biogeochemical model of soil organic matter decomposition. We review experimental and observational studies to assess whether—and in what direction—microbial allocation to exoenzyme production adapts with warming. We then evaluate the global consequences of this trait adaptation by comparing projections of soil carbon stocks and respiration with and without eco‐evolutionary optimization under a representative climate‐warming scenario. Finally, we compare our predictions of the temperature sensitivity of soil organic carbon decomposition to those of the Q10 formulation commonly used in Earth system models.

## 2 Methods

### 2.1 Biogeochemical Model

**FIGURE 1:**
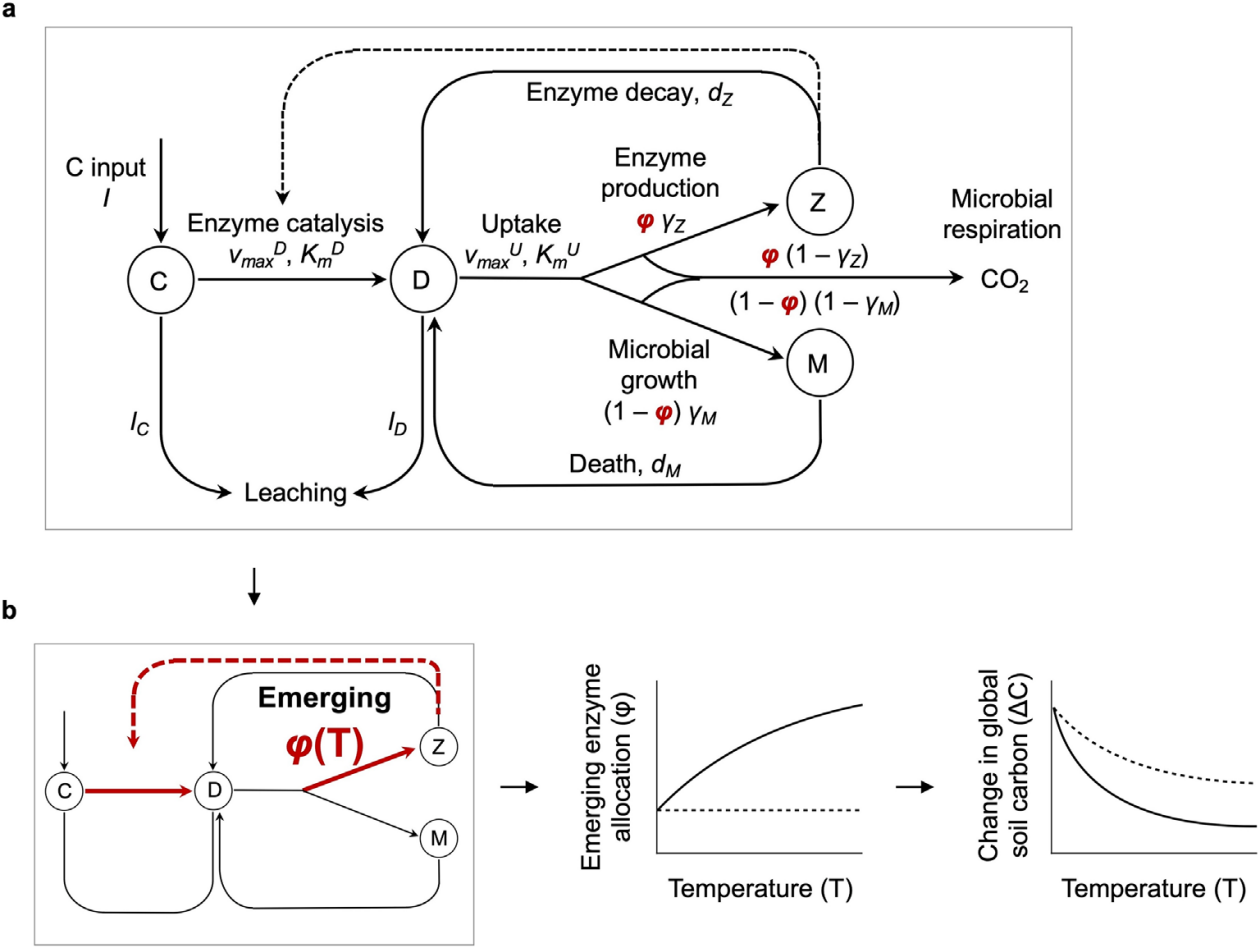
Microbe‐enzyme model (a) without and (b) with eco‐evolution. (a) The baseline model includes soil organic carbon (*C*), dissolved organic carbon used as a substrate for microbes (*D*), microbial biomass (*M*), and extracellular enzymes (*Z*), with *φ* representing the allocation of microbial carbon resources to extracellular enzymes. (b) In the eco‐evolutionary model, the temperature dependence of enzyme allocation emerges from the model. If enzyme allocation increases with temperature (solid line), warming leads to greater enzyme production and increased soil carbon loss. In contrast, in the baseline model (dashed line), enzyme allocation does not respond to temperature.

Our baseline microbe‐enzyme model (Figure 1a) is a second‐order model of soil organic carbon (SOC) dynamics that extends the model of (Allison et al. 2010) (hereafter the Allison–Wallenstein–Bradford (AWB) model) by explicitly representing the focal trait (carbon allocation to exoenzyme production) and the physiological tradeoff with microbial growth (Malik et al. 2020). The model state variables are SOC (C), dissolved organic carbon (DOC, D in the model), microbial biomass carbon (M) and an explicit enzyme carbon mass (Z). SOC increases with litter input (*I*) and decreases with enzyme decomposition, which is assumed to follow a Michaelis–Menten function of the enzyme pool. A leaching term (*e*_*C*_) accounts for small amounts of carbon lost from the system due to abiotic processes. SOC thus changes through time according to the following equation.

(1) $$\frac{\mathrm{dC}}{\mathrm{dt}}=I-e_{C}C-\frac{v_{\max}^{D}C}{K_{m}^{D}+C}Z.$$

DOC receives the product of SOC decomposition, as well as the product from the recycling rate of dead microbes (*d*_*M*_) and inactive enzymes (*d*_*Z*_). We did not include litter input to the DOC pool, represented in the AWB model, because the steady states are not sensitive to it (see sensitivity analysis in Table S2) and it simplifies the analytical treatment of the model. DOC decreases with leaching (*e*_*D*_) and microbial uptake and respiration, where it is assumed that microbial cells have a limited amount of transporters; hence another Michaelis–Menten process is represented by the last term in the following equation.

(2) $$\frac{\mathrm{dD}}{\mathrm{dt}}=\frac{v_{\max}^{D}C}{K_{m}^{D}+C}Z+d_{M}M+d_{Z}Z-e_{D}D-\frac{v_{\max}^{U}D}{K_{m}^{U}+D}M.$$

Microbial biomass (M) turns over with rate *d*_*M*_ and grows proportionally to DOC uptake and a growth efficiency term that accounts for the loss to respiration due to the energetic cost of biomass production (*γ*_*M*_), minus a fraction, *φ*, of biomass allocated to exoenzyme production (*φ*)

(3) $$\frac{\mathrm{dM}}{\mathrm{dt}}=\left(1-\varphi \right)\gamma_{M}\frac{v_{\max}^{U}D}{K_{m}^{U}+D}M-d_{M}M$$

*φ*, highlighted in red in Figure 1, is the critical phenotypic trait that we attempted to compare with empirical data in the Results section. Finally, the enzyme pool (Z) grows with microbial uptake of carbon and subsequent allocation to enzyme production and declines with enzyme degradation:

(4) $$\frac{\mathrm{dZ}}{\mathrm{dt}}=\varphi \gamma_{Z}\frac{v_{\max}^{U}D}{K_{m}^{U}+D}M-d_{Z}Z$$

The resource allocation trade‐off is parameterized with *φ*, the fraction of DOC uptake allocated to exoenzyme production, and (1‐*φ*) the fraction allocated to growth. Soil microbial respiration is the residual carbon that leaves the D pool but is neither assimilated into microbial biomass M nor allocated to enzymes Z during microbial growth (*γ*_*M*_) and enzyme production (*γ*_*Z*_) (equal to $\frac{v_{\max}^{U}D}{K_{m}^{U}+D}M\left[\varphi \left(1-\mathrm{\gamma Z}\right)-\left(1-\varphi \right)\left(1-\mathrm{\gamma M}\right)\right]$). Parameter units and values are detailed in Table S1, and steady states are detailed in Note S1.

### 2.2 Temperature Dependence

At the molecular and cellular level, the effect of warming on microbial processes is mediated by the temperature sensitivity of intra‐ and extracellular enzymatic activity (Burns et al. 2013; German et al. 2012; Wallenstein et al. 2009). In a baseline “kinetics only” scenario, microbial uptake parameters (maximum uptake rate, $v_{\max}^{U}$, half‐saturation constant, $K_{m}^{U}$) and exoenzyme kinetic parameters (maximum decomposition rate, $v_{\max}^{D}$, half‐saturation constant, $K_{m}^{D}$) increase with temperature according to the Arrhenius relationship (Davidson and Janssens 2006; Hochachka and Somero 2002):

(5) $$v_{\max}^{D}=v_{0}^{D}e^{-\frac{E_{v}^{D}}{R\left(T+273\right)}}$$

(6) $$K_{m}^{D}=K_{0}^{D}e^{-\frac{E_{K}^{D}}{R\left(T+273\right)}}$$

(7) $$v_{\max}^{U}=v_{0}^{U}e^{-\frac{E_{v}^{U}}{R\left(T+273\right)}}$$

(8) $$K_{m}^{U}=K_{0}^{U}e^{-\frac{E_{K}^{U}}{R\left(T+273\right)}}$$

Other temperature scenarios are presented in Note S2.

### 2.3 Eco‐Evolutionary Framework

In the original AWB model, the production of enzymes by microbes was a user‐selected external parameter, insensitive to warming (Figure 1a). Here, we use an eco‐evolutionary framework to prognostically simulate how warming‐induced acceleration of enzyme kinetics changes exoenzyme production. Thus, in our simulations, exoenzyme production is a predicted outcome of an endogenous eco‐evolutionary optimization process (Figure 1b). We use evolutionary game theory extended to general ecological scenarios (Geritz et al. 1998; Metz et al. 1992) to predict the strength and direction of selection on trait *φ* and its evolutionarily stable value, φ*. In this framework, the interaction between ecological and evolutionary processes is captured by the competition between a “resident strategy” (wild type) and alternate strategies (“mutants”) within a set of feasible phenotypes. In a given environment (e.g., at a given temperature), the optimal phenotype is identified as an evolutionarily stable strategy (ESS), i.e., a phenotype that, when resident, no mutant can invade. Here the set of feasible phenotypes is the range (*φ*_min_, *φ*_max_) at a given temperature, for which a stable ecosystem equilibrium with nonzero microbial biomass exists.

To model the competition effect of a resident phenotype, *φ*_res_, on the population growth of a mutant phenotype, *φ*_mut_, we extend the baseline microbial enzyme model written for a single trait value (Equation 3). To account for the local nature of the interaction between cells with different phenotypes, we introduce a function (hereafter denoted by *c*) of the difference between trait values *φ*_res_ and *φ*_mut_ to measure how local decomposition by mutant and resident cells differs from “mean field” (average) decomposition by resident cells only. Thus, for given *C*, *D*, *Z*, the growth of the mutant population is governed by:

(9) $$\frac{dM_{\mathrm{mut}}}{\mathrm{dt}}=\left(1-\varphi_{\mathrm{mut}}\right)\gamma_{M}\frac{v_{\max}^{U}\left(1+c\left(\varphi_{\mathrm{mut}}-\varphi_{\mathrm{res}}\right)\right)D_{\mathrm{res}}}{K_{m}^{U}+\left(1+c\left(\varphi_{\mathrm{mut}}-\varphi_{\mathrm{res}}\right)\right)D_{\mathrm{res}}}M_{\mathrm{mut}}-d_{M}M_{\mathrm{mut}}$$

where *D*_res_ is the equilibrium *D* predicted by the ecosystem model for the resident phenotype *φ*_res_. Here function *c* satisfies *c*(0) = 0, *c*(*z*) > 0 if *z* > 0 and *c*(*z*) < 0 if *z* < 0. Thus, each microbe has access to DOC partly as a public good and partly as a private good (Driscoll and Pepper 2010). The public good part results from the diffusion of exoenzymes. The private good part results from local decomposition at the microscopic scale by exoenzymes that the cells produce themselves. A mutant cell that invests more in exoenzymes has access to more DOC than the average resident cell because the cell's private good is greater, whereas all cells share the same public good. In a spatially implicit model like ours, diffusion is not modeled explicitly, but its effect on the accessibility of DOC to a mutant strain can be parameterized by putting mutant cells at a competitive advantage for DOC if the mutant phenotype invests more in exoenzyme production than the resident phenotype, or at a competitive disadvantage if the mutant phenotype invests less. This parameterization is achieved with the function *c* in Equation 9, where *c* < 0 when *φ*_mut_ < *φ*_res_ and *c* > 0 when *φ*_mut_ > *φ*_res_. This approach is consistent with the mathematical construction and numerical analysis of a spatially explicit model of resident‐mutant local interaction that accounts for soil diffusion (Abs et al. 2020).

Mutant relative fitness *s*(*φ*_*mut*_, *φ*_*res*_) is given by the mutant population growth rate per unit biomass:

(10) $$s\left(\varphi_{\mathrm{mut}},\varphi_{\mathrm{res}}\right)=\left(1-\varphi_{\mathrm{mut}}\right)\gamma_{M}\frac{v_{\max}^{U}\left(1+c\left(\varphi_{\mathrm{mut}}-\varphi_{\mathrm{res}}\right)\right)D_{\mathrm{res}}}{K_{m}^{U}+\left(1+c\left(\varphi_{\mathrm{mut}}-\varphi_{\mathrm{res}}\right)\right)D_{\mathrm{res}}}-d_{M}$$

The selection gradient is then obtained by taking the first‐order derivative of Equation 10, the mutant relative fitness, with respect to the mutant trait:

(11) $$\nabla s\left(\varphi \right)={\left[\frac{\partial s\left(\varphi_{\mathrm{mut}},\varphi_{\mathrm{res}}\right)}{\partial \varphi_{\mathrm{mut}}}\right]}_{\varphi_{\mathrm{mut}}=\varphi_{\mathrm{res}}=\varphi }=\frac{d_{M}}{1-\varphi }\left(\left(1-\varphi -\frac{d_{M}}{v_{\max}^{U}\gamma_{M}}\right)c_{0}-1\right)$$

where *c*_0_ = *c′*(0) measures competition asymmetry between phenotypes, i.e., the local competitive advantage to stronger exoenzyme producers. Note that by definition of function *c*, we always have *c*_0_ > 0. Variation in *c*_0_ may be caused by different soil diffusion properties, due to e.g., physical texture or moisture.

Trait values that nullify the selection gradient (where $\nabla s\left(\varphi \right)$= 0) are called “evolutionary singularities”. An evolutionary singularity can be attractive or repelling, and invadable or noninvadable (ESS). Evolutionary singularities that are attractive and noninvadable represent eco‐evolutionary optima that can be reached by the adaptation process; evolutionary singularities that are attractive and invadable can lead to evolutionary branching (Geritz et al. 1998). In a given environment (fixed parameters, constant temperature) there is at most one evolutionary singularity given by defining φ* as the value of *φ* that makes $\nabla s\left(\varphi \right)$= 0:

(12) $$\varphi^{*}=1-\frac{d_{M}}{\gamma_{M}v_{\max}^{U}}-\frac{1}{c_{0}}$$

Existence of φ* > 0 requires $\frac{d_{M}}{v_{\max}^{U}\gamma_{M}}<1$ and $c_{0}>\frac{1}{\left(1-\frac{d_{M}}{v_{\max}^{U}\gamma_{M}}\right)}$. Thus, the (cooperation) trait *φ* can evolve above zero only if the local competition advantage to stronger enzyme producers is large enough. The condition for φ* to be evolutionarily stable is *c″*(0) < 2 c_0_^2^, which means biologically that the competition advantage needs to be toward lowering the gain for lower‐producers rather than increasing the gain for higher‐producers. No other condition than existence is required for φ* to always be convergent. Here we assume that function *c* is such that φ* is evolutionarily stable and attractive.

### 2.4 Literature‐Based Qualitative Empirical Validation

To evaluate whether the model‐predicted eco‐evolutionary response of increased enzyme allocation with warming is supported by empirical evidence, we reviewed 13 unique studies that included nine warming experiments, seven incubation assays, two meta‐analyzes synthesizing 56 and 78 warming studies, and one latitudinal metagenomic survey (Table S4). Several studies combined multiple approaches (e.g., both warming experiments and enzyme assays). These studies encompassed warming treatments ranging from +0.5°C to +18°C, durations from 2 months to 50 years, and diverse methodological approaches, including respiration measurements, enzyme assays, community structure analyzes (16S, fungal: bacterial ratio, taxonomic profiling), and functional analyzes (metagenomics, metatranscriptomics). Notably, respiration and enzyme activity data alone—without genomic analyzes or isolate‐based assays—cannot reliably distinguish microbial adaptation from shifts in enzyme temperature sensitivity or in enzyme turnover. For this reason, we treated each meta‐analysis as a single, aggregate line of evidence, rather than counting individual studies within them, to avoid over‐representing support from less mechanistically specific data sources.

### 2.5 Simulations at the Global Scale

We evaluated the effect of microbial eco‐evolution on global soil carbon and respiration under climate projections for the 21st century. To this end, we calibrated the AWB model against a vertically solved version of MIMICS (Wieder et al. 2013), a model widely used in contemporary ecosystem modeling frameworks (He, Abramoff, et al. 2024; He, Abs, et al. 2024; Pierson et al. 2022; Schimel 2023; Tao et al. 2023). Wieder et al. (2013) calibrated parameters to reproduce observations of current soil carbon stocks (Fao and Isric 2012). Thus, our global simulations used a parameterization that was extensively validated by current soil carbon data at both local and global scales. We used the soil surface temperature projections of the Community Climate System Model (CCSM4) for the Representative Concentration Pathway 8.5 (RCP8.5) (Worley et al. 2011) as forcing data to simulate soil carbon stocks and respiration over approximately 8000 1° grid points across continental scales. We ran two versions of the AWB model, one in which microbial allocation to exoenzyme production is fixed, and one in which eco‐evolutionary optimization of microbial allocation occurs as warming proceeds. To compare soil carbon predictions with and without eco‐evolutionary optimization, we initialized both versions of the model with enzyme allocation optimized to the local 2010 temperatures (Figure S1), assuming that soil carbon stocks were locally at an equilibrium in 2010 (Figure S2).

We obtained global projections of soil carbon stocks and respiration by computing the steady state values at a given warming level across terrestrial continents. Input soil temperature projections were obtained from version 4 of the CCSM4 for the RCP8.5 from 2005 to 2100 (publicly available online at https://www.cesm.ucar.edu/models/ccsm4.0/) (Worley et al. 2011). We averaged those projections over the last year of each decade to calculate the local steady states shown in all the figures. For all sites where the microbial activity is positive, the soil carbon stock was estimated as the sum of the steady states of SOC and microbial biomass (in units of carbon mass per m^2^ per centimeter of soil depth). To evaluate the effect of optimization, we calculated the sum of changes in soil carbon stock and respiration of every decade between 2010 and 2100. For the predictions obtained with alternative scenarios of microbial temperature sensitivity, we used equations and parameters from Allison et al. (2010) and Hagerty et al. (2014) (see equations in Note S2). For the predictions obtained with spatial and temporal variability in litter input, we used the litter projections from CESM (same model source as for temperature), and averaged them as well over the last year of each decade at each site. Finally, for the predictions obtained with spatially variable temperature sensitivity of enzyme kinetics, we used the five sets of sensitivity parameters obtained by German et al. (2012). We divided continents into five environmental clusters based on temperature and moisture (Figure S3) and used the corresponding set of enzyme temperature sensitivity parameters from German et al. (2012) (Table S3).

### 2.6 Comparison With a Q10‐Based Reference Model

To evaluate how eco‐evolutionary optimization alters predicted temperature responses of soil carbon stocks, we compared our results to those of a classical Q10 model. Earth System Models (ESM) typically represent warming‐induced increases in decomposition rates using a Q10 formulation, in which the decay rate increases exponentially with temperature

(13) $$\frac{\mathrm{dC}}{\mathrm{dt}}=I-r_{0}Q10^{\frac{T-T_{0}}{10}}C$$

with a typical value of *Q*10 equal to 2 (Wu et al. 2021). While *Q*10 is usually used to represent the temporal sensitivity of respiration to temperature, our study focuses on steady states, where respiration equals litter input and is thus temperature‐independent (since litter input is held constant across temperatures in our baseline configuration). Instead, temperature sensitivity appears in the equilibrium decay rate and carbon stocks. To allow meaningful comparison, we applied the *Q*10 formulation to these steady‐state variables, parameterized it to match the AWB model at 10 °C, and compared it to predictions from AWB with and without evolution.

## 3 Results

### 3.1 Eco‐Evolutionary Optimization Amplifies Warming‐Induced Global Soil Carbon Loss

**FIGURE 2:**
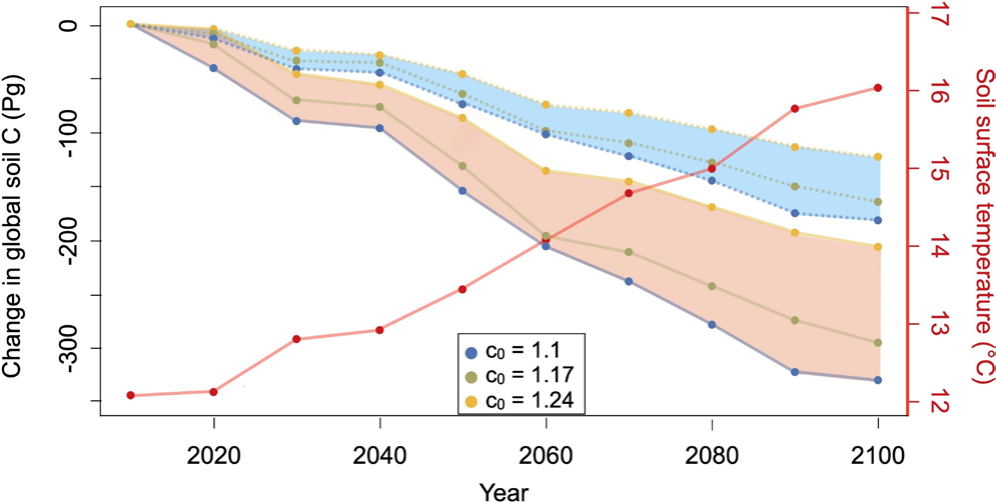
Projections of global soil carbon stocks from 2010 to 2100. Temporal change in global total soil carbon without (dashed lines, blue shade) and with (solid lines, orange shade) microbial eco‐evolutionary optimization for three values of the competitive advantage model parameter (*c*_*0*_) (left y axis). Lower *c*_*0*_ leads to lower enzyme allocation (*φ*), resulting in higher initial soil carbon stocks, but also in higher temperature sensitivity of enzyme allocation, hence in higher soil carbon losses under warming, by 2100. Temporal change in global average surface soil temperature between 2010 and 2100 from the RCP8.5 scenario with the CCSM4 model (right y axis, red). Global soil carbon stock is calculated every decade between 2010 and 2100, as the sum of local soil carbon stock across all sites. Parameter values are given in Table S1.

The original AWB model predicted a soil carbon loss of approximately 150 Pg by 2100 under the RCP8.5 warming scenario (Figure 2, blue shade), falling within the large range (−300 Pg, +20 Pg) given by current Earth system models with no explicit description of soil microbes (Wieder et al. 2013). When eco‐evolutionary optimization of microbial exoenzyme production in response to warming was incorporated, the projected global soil carbon loss by 2100 nearly doubled (Figure 2, orange shade). Soil heterotrophic respiration (SHR) followed a similar pattern: the original AWB model projected an increase of SHR of approximately +2.2 PgC yr^−1^ between 2010 and 2100, while the eco‐evolutionary model predicted a larger increase of about +3.5 PgC yr^−1^ (Figure S4).

These higher carbon losses and SHR rates occur because our eco‐evolutionary model predicts that warming selects for higher enzyme production, leading to a higher decomposition rate of SOC. The robustness of this finding is shown by a sensitivity analysis in which we vary a key parameter of eco‐evolutionary optimization, the local competitive advantage, denoted by *c*_*0*_, that higher exoenzyme producers have over lower‐producers (Figure 2). This parameter is associated with a function (Equation 9) that implicitly represents the effects of spatial heterogeneities associated with resource diffusion in soils and with microbial interactions. It can vary between low values (close to 0), in which case there is no advantage to local enzyme producers (hence nonenzyme‐producing microbes act as cheaters that reap the benefit of enzymes without producing them), to very large values, where microbial enzyme producers capture most of the benefit of their production (Equations (11), (12)).

To better understand the simulation results, we analyzed the relationship between the eco‐evolutionary optimum, which can be derived analytically, and temperature. The eco‐evolutionary optimum is an increasing function of microbial growth efficiency, maximum uptake rate, and intensity of competition between different strains, and a decreasing function of mortality (Equation 12). Focusing on the temperature dependence of the maximum uptake rate, which reflects the well‐known biochemical dependence of enzyme kinetics on temperature (van't Hoff 1884), we find that the optimal exoenzyme allocation responds to temperature only through the positive temperature dependence of the maximum uptake rate (Equations (5), (6), (7), (8)). As a consequence, optimal exoenzyme allocation is an increasing function of temperature (further illustrated in Figure 4b).

### 3.2 Effect of Microbial Eco‐Evolutionary Optimization Is Stronger in Cold Regions

**FIGURE 3:**
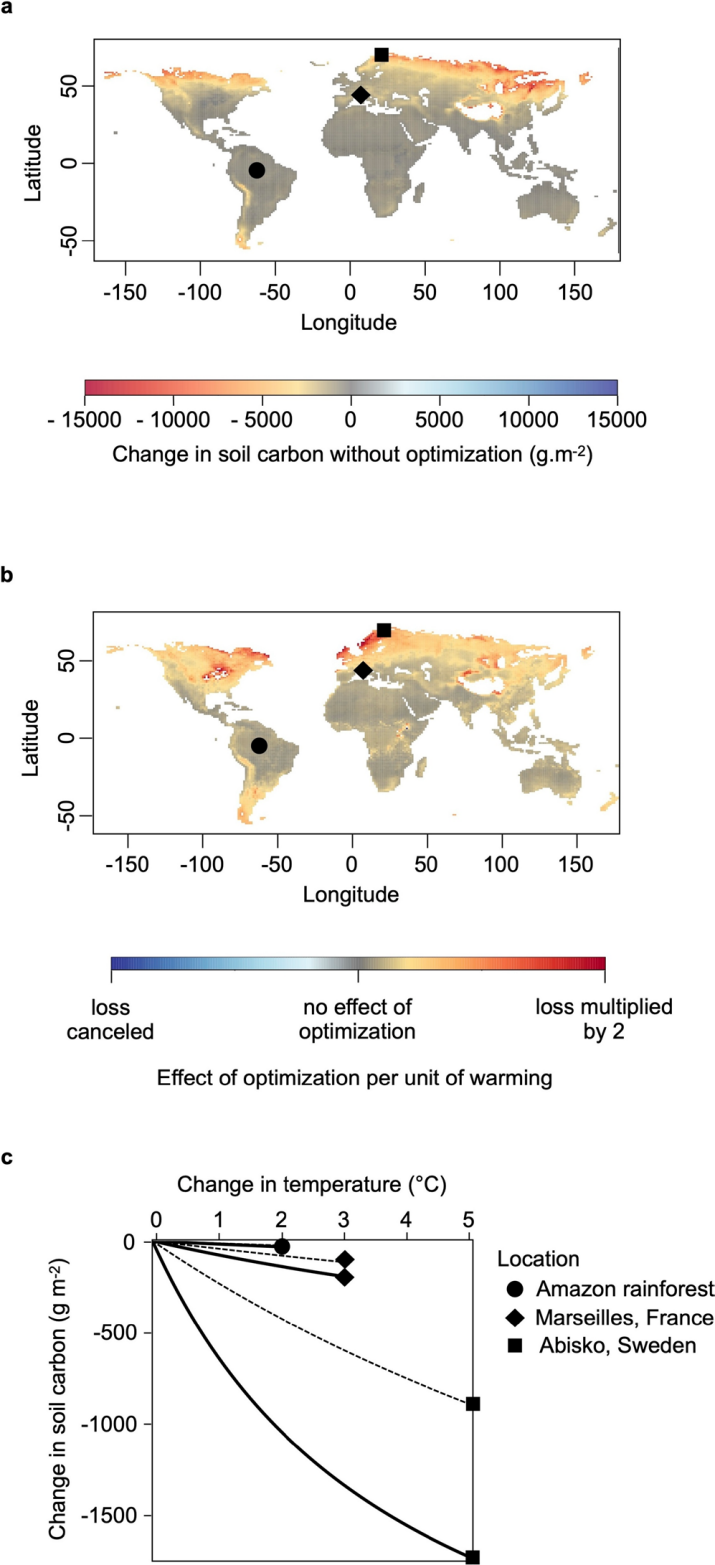
Model projections under the RCP8.5 soil temperature warming scenario. (a) Changes in soil carbon between 2010 and 2100 without optimization. (b) Effect of eco‐evolutionary optimization per unit of warming between 2010 and 2100. The effect of optimization is the difference between the model runs with and without optimization. (c) Sensitivity of soil carbon responses to changes in mean annual temperature (MAT) without (dashed lines) and with (solid lines) eco‐evolutionary optimization at three sites, taken to represent the distinct responses in cold (Abisko, Sweden, Arctic wetland, circle), moderate (Marseilles, France, Mediterranean shrubland, diamond) and warm (Manaus, Brazil, tropical rainforest, square) regions. Note that in extremely high latitude regions (Antarctica, high Arctic) the absence of predictions is due to insufficient soil or model instability where temperatures are too low to support meaningful microbial activity. We used the intermediate value of *c*_*0*_ = 1.17. Parameter values are given in Table S1. Map lines delineate study areas and do not necessarily depict accepted national boundaries.

This fundamental pattern of optimal allocation to exoenzyme production increasing with temperature provides a basis for predicting and understanding regional patterns of eco‐evolutionary responses of soil carbon to climate warming. Starting with the global pattern of soil carbon loss (Figure 3), we found that soil carbon loss, even in the non‐eco‐evolutionary scenario, was greatest at high latitudes (Figure 3a) and that eco‐evolutionary optimization amplified this trend (Figure 3b).

To further analyze the regional patterns of warming‐induced soil carbon loss, we examined the sensitivity of soil carbon responses to temperature with and without eco‐evolutionary optimization at three example sites across latitude (Figure 3c). Without eco‐evolutionary optimization, soil carbon loss for a given increase in temperature is higher in cold regions because of the non‐linearity of the Arrhenius temperature sensitivity of enzyme kinetics, a trend that is further amplified (by a factor of ~2.5 in the Arctic, but negligibly in the tropics) with eco‐evolutionary optimization (Figure 3c). Thus, optimization of microbial enzyme allocation has little to no effect at low to mid latitudes (central and Northern South America, Africa, SE Asia, Australia), but doubles the loss of soil carbon in northern temperate to high latitudes (e.g., continental US) and triples soil carbon loss in boreal, arctic, and alpine cold regions (Scandinavia, NE Canada, South Chile) (Figure 3b). We find the same trend for SHR, with eco‐evolutionary optimization having little to no effect at low to mid latitudes, but doubling to tripling SHR in northern temperate to high latitude regions (Figure S5).

**FIGURE 4:**
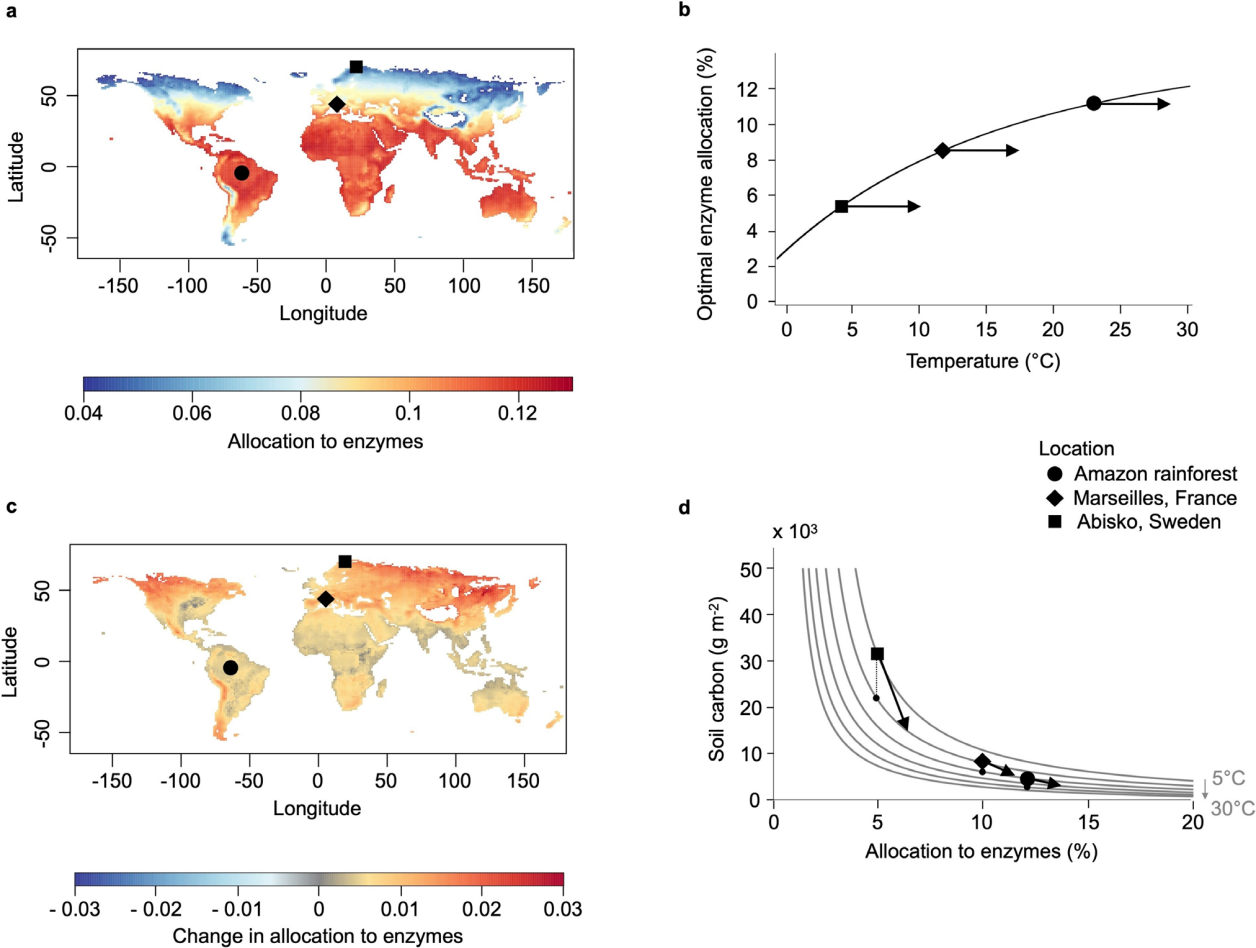
Predicted optimal exoenzyme allocation (φ*) and joint effects of warming and exoenzyme allocation optimization on soil carbon. (a) Global distribution of φ* in 2010 predicted from Equation (12) using the intermediate value of *c*_*0*_ = 1.17. (b) Sensitivity of φ* to temperature. Arrows show the effect of hypothetical identical 5°C increases, with initial temperature from 2010 at the same Arctic, Temperate and Tropical sites identified in Figure 3 (4.2°C, 12.6°C and 23.6°C). (c) Global distribution of change in φ*predicted by the model with eco‐evolutionary optimization and RCP8.5 soil temperature predictions for 2010 and 2100. (d) Modeled sensitivity of soil carbon to both *φ* and temperature. 2010 values and responses to a hypothetical identical 5°C increase are shown for the three sites identified in Figure 3. Arrows show the effect without (dashed lines to black circle; enzyme temperature kinetics only) and with (solid arrows) eco‐evolutionary optimization, which involves an increase in *φ* that amplifies the kinetics only soil carbon loss. Parameter values are given in Table S1. Map lines delineate study areas and do not necessarily depict accepted national boundaries.

The effect of microbial eco‐evolutionary optimization causes a stronger amplification of warming‐induced soil carbon loss at high latitudes for three reasons. First, the climate‐warming scenario used as forcing data has a polar amplification of warming, reaching up to +5°C north of 60° N compared to a global mean warming of only +3.2°C by 2100 (Figure S6). This enhanced warming at high latitudes drives stronger microbial responses, whether eco‐evolutionary optimization is included or not. However, the effect of optimization per unit of warming is still the highest at high latitudes (Figure 3b), so polar amplification alone cannot explain the results shown in Figure 4. The second reason is that, although exoenzyme allocation is lower at low temperatures (Figure 4a), optimal exoenzyme allocation is a nonlinear convex increasing function of temperature (Figure 4b). Therefore, for a given increase in temperature, optimal exoenzyme allocation increases more at low than at high temperatures. As a result of both the latitudinal gradients of warming and the higher temperature sensitivity of exoenzyme allocation in cold regions, optimal exoenzyme allocation increases more at high latitudes (Figure 4c). Finally, soil carbon is a nonlinearly decreasing function of exoenzyme allocation (Figure 4d). For the same variation in exoenzyme allocation, soil carbon decreases more at locations where exoenzyme allocation is initially low. This also explains why the predicted change in soil carbon without optimization differs between *c*_0_ values. Lower *c*_0_ values lead to lower exoenzyme allocation, and therefore higher sensitivity of soil carbon to warming.

### 3.3 Effect of Microbial Trait Optimization Is Robust to Parameters and Climate Scenarios

To assess the robustness of our results, we examined different scenarios of temperature sensitivity, litter response, and enzyme diversity. Even when microbial growth efficiency (*γ*_*M*_) or mortality (*d*_*M*_) is sensitive to temperature (Allison et al. 2010; Hagerty et al. 2014), eco‐evolutionary optimization of exoenzyme allocation generally still results in amplification of soil carbon losses (Figure S7). As a notable exception, if the sensitivity of microbial mortality to temperature is high, there is a decrease in exoenzyme allocation with warming (Figure S8c) because the process of fitness maximization trades off against a larger increase in mortality with a large investment in microbial growth, leaving less carbon for exoenzyme production (Equation 12).

It is worth noting that warming‐induced increases in plant productivity in some regions and associated increases in litter inputs to the soil are an independent mechanism that was not accounted for in our simulations and could partially compensate for (or even reverse) the soil carbon losses from increased soil carbon decomposition. When projected changes (usually increases) in litter inputs from CCSM4 RCP8.5 are combined with changes in decomposition from AWB (projections taken from the same CESM model and scenario as for surface soil temperature), total global soil carbon was predicted to slightly increase by 2100 in the non‐eco‐evolutionary scenario (Figure S9, black dashed line). However, with eco‐evolutionary optimization, these gains due to increased litter production are offset by higher decomposition rates (Figure S9, black solid line).

Finally, we consider the potential effects of spatial variation in enzyme kinetics around the globe. We use measurements of enzyme kinetics in different biomes along a latitudinal gradient (German et al. 2012) to create a map of enzyme kinetic variation. We find that spatial variation in enzyme kinetics increases the effect of eco‐evolutionary optimization (Figure S10), while integrating variation both in litter and enzyme kinetics may reduce the effect of eco‐evolutionary optimization (Figure S11).

### 3.4 Empirical Studies Mostly Support Increased Microbial Enzyme Allocation With Warming

We examined whether empirical studies support our model prediction that warming selects for increased microbial enzyme allocation. Among the 13 studies we reviewed, nine supported our prediction that microbial communities adapt to warming by increasing enzyme production—six via eco‐evolutionary adaptation (e.g., community shifts, genetic changes) and three via physiological response (e.g., gene expression changes)—while three studies contrasted with our predictions by reporting no effect of warming on enzyme production, though with varying levels of confidence.

For instance, the soil warming experiment by Guo et al. (2020) found that warming upregulated 58%–80% of biogeochemical cycling genes, including those involved in lignin, cellulose, and hemicellulose degradation. This result strongly supports our prediction, as metagenomics provides high genetic resolution of microbial communities and captures metabolic potential under a range of conditions. In contrast, studies from the 30‐year Harvard Forest warming experiment produced contradictory results, potentially due to phase‐dependent warming responses (Melillo et al. 2017), indirect effects of SOC depletion (Domeignoz‐Horta et al. 2023), and horizon mixing in bulk soil samples, which may obscure contrasting responses occurring in different soil layers (Pold et al. 2016).

### 3.5 The Q10 Model Fails to Capture Soil Carbon Accumulation at Low Temperatures

**FIGURE 5:**
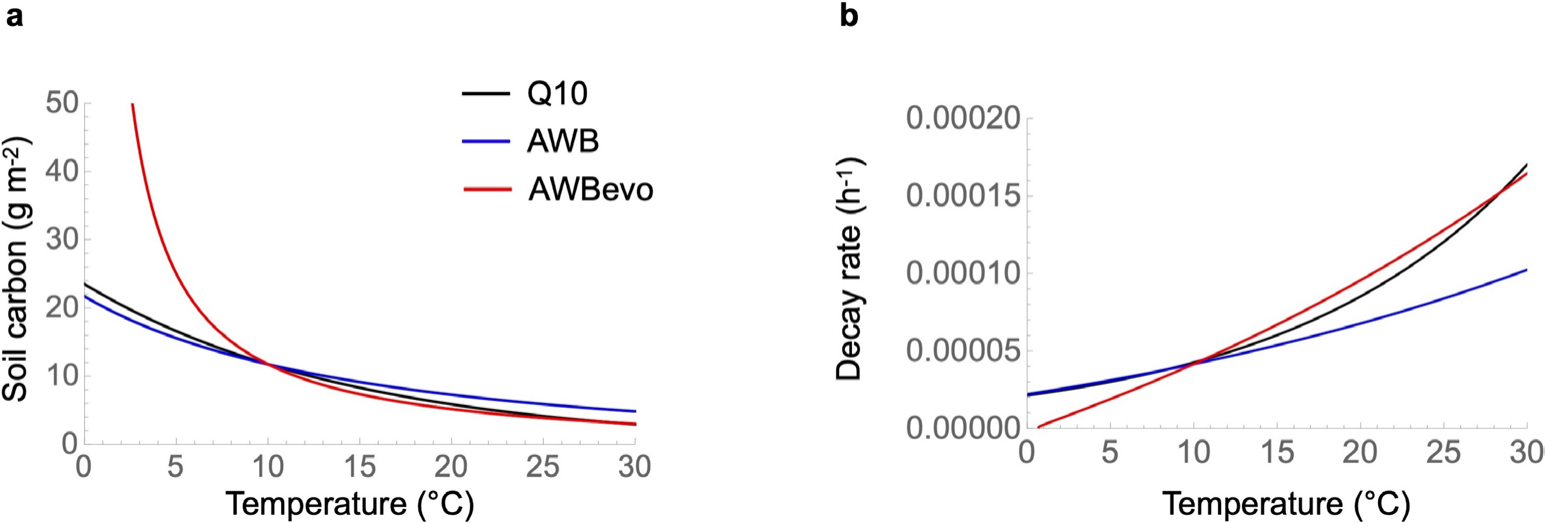
Comparison of the emerging relationship between equilibrium SOC (a) or decay rates (b) and temperature. Lines correspond to the AWB model without evolution (blue), AWB with evolution (red), and the ‘*Q*10 model’ with *Q*10 = 2.

We compared the temperature sensitivity of equilibrium soil carbon predicted by AWB with evolution to that of a classical Q10 model and of AWB without evolution, and found that only AWB evo with evolution predicts soil carbon accumulation at low temperatures (Figure 5). AWB with evolution shows a markedly greater temperature sensitivity at low temperatures (Figure 5a), because only in this model with evolution can microbes adapt their enzyme allocation to temperature; at low temperatures, they adapt by allocating very little to enzyme production, which reduces the decay rate to near zero (Figure 5b) and leads to the accumulation of carbon stocks (Figure 5a).

## 4 Discussion

**FIGURE 6:**
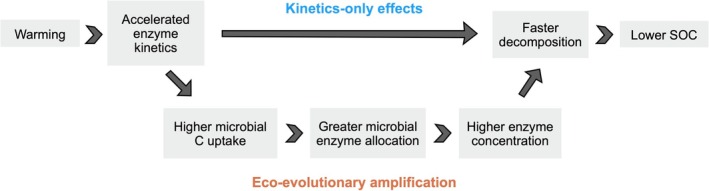
How temperature affects the eco‐evolutionary optimization of microbial enzyme allocation in response to warming. Eco‐evolutionary optimization results in even faster decomposition and ultimately greater SOC loss than without eco‐evolutionary optimization.

Microbial communities are expected to respond to climate change through a variety of ecological and evolutionary processes. In this study, we asked whether microbial eco‐evolution could substantially alter global soil carbon projections over the next century under climate warming, and whether this effect would be spatially uniform or vary nonlinearly with temperature. Using adaptive dynamics optimization theory within a widely used ecological model of organic matter decomposition, we found that the eco‐evolutionarily optimal allocation to exoenzymes increases with increasing temperature, driven by the positive temperature dependence of microbial maximum uptake rate. This microbial optimization strategy amplifies the effect of the baseline kinetic response, increasing soil carbon turnover with warming (Figure 6). Accounting for eco‐evolutionary optimization resulted in 1.8 times greater global soil carbon loss between 2010 and 2100. Importantly, this effect is not spatially uniform: the amplification of carbon loss is much stronger in cold regions due to the nonlinear temperature sensitivity of optimized microbial enzyme allocation. Finally, in addition to these warming responses, the eco‐evolutionary model also predicts higher soil carbon stocks at low temperatures—consistent with empirical observations of greater SOC accumulation in cold climates (García‐Palacios et al. 2024; Hartley et al. 2021), which Q10‐based models currently used in Earth system models fail to capture.

### 4.1 Global Carbon Cycling Patterns: Comparison With Previous Model Predictions

Our projected range of change in global soil carbon stocks (−100 to −300 Pg C) falls within estimates reported in the literature. For example, Wieder et al. (2013) simulated SOC changes ranging from 0 to −300 Pg C, depending on assumptions about microbial adaptation. For soil heterotrophic respiration (SHR), our global estimate of 37 PgC yr^−1^ for the present day is consistent with the range 33–46 PgC yr.^−1^ reported by Ciais et al. (2021). Nissan et al. (2023) projected a + 16.8 PgC yr^−1^ increase in global SHR under combined temperature and moisture effects. Although they did not quantify the individual contributions, they identified moisture as the dominant driver. Based on this, we infer that the temperature contribution is below 8 PgC yr.^−1^ but still substantial—consistent with the magnitude of our warming‐only projection.

### 4.2 Underlying Mechanisms Driving SOC and SHR Responses

While the magnitudes of our projected changes in SOC and SHR are consistent with prior studies, the underlying mechanisms differ. Melillo et al. (2017) observed a strong initial SHR response to warming, followed by a long‐term decline in temperature sensitivity. This shift has been attributed to “acclimatization” (Luo et al. 2001) or “thermo‐adaptation” (Bradford 2013), although it may also reflect substrate limitation or eco‐evolutionary responses—or a combination of these, as Melillo et al. suggested. Our model explicitly accounts for both substrate dynamics and microbial trait adaptation. It predicts that increased decomposition under warming arises not only from faster enzyme kinetics but also from an eco‐evolutionary increase in exoenzyme production. As for SOC, Wieder et al. (2013) also projected greater SOC loss under warming if microbial communities could adapt. In their model, adaptation was assumed to allow microbes to maintain pre‐warming maintenance costs, thereby avoiding a warming‐induced decline in carbon use efficiency (CUE) and maintaining higher rates of microbial decomposition. However, this assumption was not derived from empirical evidence or a formal evolutionary framework.

In contrast, our model derives the increase in exoenzyme allocation directly from microbial competition and evolutionary dynamics. To understand why warming increases allocation to enzymes in our model, it is important to note that at the individual scale, microbial life history strategies must balance a fundamental trade‐off between investment in growth and investment in resource acquisition (Malik et al. 2020). Enzymes underlying resource acquisition function extracellularly and thus form a public good, which sets the stage for cheaters, i.e., microbes that produce less exoenzymes but take up carbon monomers produced by other microbes' enzymes. In our model, the stability of exoenzyme production in the face of cheating, that is, why cheaters do not become the dominant type in a community, is rooted in the spatial heterogeneity that the model represents implicitly (Equation 9) by making monomer accessibility to a given phenotype increase with that phenotype's investment in exoenzymes. By driving faster enzyme kinetics, and in particular the uptake kinetics, warming decreases the likelihood of successful cheating and therefore increases the return on investment in exoenzymes. As a result, microbes that invest more in exoenzyme production (and less in biomass production) have a higher net growth rate. Eco‐evolutionary optimization thus leads to microbial communities with higher allocation to exoenzymes in response to warming. Our literature review (Table S4) highlighted support for this prediction in several studies, particularly those employing metagenomics and metatranscriptomics (e.g., Guo et al. 2020). However the Harvard Forest experiment suggest more complex underlying mechanisms: microbial adaptation appears to be dynamic over time (Melillo et al. 2017), may at times be indirectly driven by SOC depletion (Domeignoz‐Horta et al. 2023), and could differ across soil horizons (Pold et al. 2016).

### 4.3 Future Directions

Our study is a first step toward understanding the role of microbial eco‐evolutionary dynamics in the global carbon cycle. One of the most pressing challenges for soil scientists is to improve climate projections while reducing uncertainty in the global soil carbon response to warming (Bradford et al. 2016). Eco‐evolutionary optimization removes uncertainty, because the critical exoenzyme allocation parameter is not fixed but instead emerges from the model (Follows et al. 2007). Additionally the recent surge in genomic data (Piton et al. 2023), combined with hierarchical modeling approaches, could help reduce uncertainty (Abs, Chase, et al. 2024). Important next steps include extending model sensitivity beyond temperature to other environmental drivers (e.g., moisture, pH) (Malik et al. 2020; Manzoni 2017), incorporating physical mechanisms such as soil structure (Borer et al. 2020), and explicitly distinguishing mechanisms of adaptation (e.g., change in demography, mutation, dispersal) (Abs, Coulette, et al. 2024). Arguably, the most critical priority is to develop the capacity to fully validate eco‐evolutionary models, which will require long‐term, multi‐scale data on microbial traits and enzyme production, alongside SOC and microbial biomass measurements (Xie et al. 2020). Future integrative empirical studies will be critical to disentangle the physiological, ecological and evolutionary mechanisms whereby soil microbial communities respond to climate warming, and to generate the data needed to design, validate and calibrate the next generation of eco‐evolutionary Earth system models.

## Author Contributions

**Elsa Abs:** conceptualization, data curation, formal analysis, investigation, methodology, validation, visualization, writing – original draft, writing – review and editing. **Scott R. Saleska:** methodology, writing – review and editing. **Steven D. Allison:** conceptualization, writing – review and editing. **Philippe Ciais:** writing – review and editing. **Yang Song:** data curation, formal analysis, investigation, validation, visualization, writing – review and editing. **Michael N. Weintraub:** data curation, investigation, writing – review and editing. **Regis Ferriere:** conceptualization, methodology, supervision, writing – review and editing.

## Conflicts of Interest

The authors declare no conflicts of interest.

## Supporting information


**Figure S1.** Global distribution of soil surface temperature in 2010. Data are taken from the RCP8.5 scenario with the CCSM4 model (note: these do not account for the eco‐evolutionary carbon cycle feedbacks, reported here, that could further affect warming). Map lines delineate study areas and do not necessarily depict accepted national boundaries.
**Figure S2.** Global distribution of soil carbon predictions in 2010. Soil carbon is the sum of the equilibrium of SOC and microbial biomass calculated with the AWB model with explicit trade‐off and optimization of allocation to enzymes to 2010 local soil temperature from the RCP8.5 scenario with the CCSM4 model. Parameter values are in Table S1. Map lines delineate study areas and do not necessarily depict accepted national boundaries.
**Figure S3.** Earth terrestrial ecoregions (a) clustered into five biomes (b) for which enzyme kinetics parameters have been measured. The five biomes, and their reference location for which enzyme kinetics were measured, are tropical forest (Costa Rica, biome 1), temperate grassland (California, biome 2), temperate deciduous forest (West Virginia, biome 3), temperate/cold coniferous forest (Maine, biome 4), and boreal forest/tundra (Alaska, biome 5). Enzyme Arrhenius parameters are in Table S3. Map lines delineate study areas and do not necessarily depict accepted national boundaries.
**Figure S4.** Projections of global CO_2_ flux from 2010 to 2100. (left y axis) Temporal change in global total CO_2_ flux without (dashed lines, blue shade) and with (solid lines, tan shade) microbial eco‐evolutionary optimization for three values of the competitive advantage model parameter (*c*_*0*_); (right y axis, red) Temporal change in global average surface soil temperature between 2010 and 2100 from the RCP8.5 scenario with the CCSM4 model. Global CO_2_ flux is calculated every decade between 2010 and 2100, as the sum of local CO_2_ flux across all sites. Parameter values (e.g., *c*_*0*_) are given in Table S1.
**Figure S5.** Model projections under the RCP8.5 soil temperature scenarios. (a) Global distribution of CO_2_ flux predictions in 2010. (b) Changes in CO_2_ flux between 2010 and 2100 without optimization. (c) Changes in CO_2_ flux between 2010 and 2100 with optimization. (d) Effect of eco‐evolutionary optimization per unit of warming between 2010 and 2100. The effect of optimization is the difference between the model runs with and without optimization. Map lines delineate study areas and do not necessarily depict accepted national boundaries.
**Figure S6.** Global distribution of change in surface soil temperature from 2010 to 2100. Predictions from the RCP8.5 scenario with the CCSM4 model. Map lines delineate study areas and do not necessarily depict accepted national boundaries.
**Figure S7.** Global projections of soil C stock from 2010 to 2100 for reference eco‐evolutionary optimization scenario (black) and for 3 other scenarios of microbial temperature sensitivity within the eco‐evolutionary model: (green) microbial growth efficiency decreases with warming, (pink) microbial mortality increases with warming, (purple) microbial mortality increases strongly with warming. Each color comes with 2 models’predictions: (dashed) no optimization, (solid) with eco‐evo optimization.
**Figure S8.** Responses of the enzyme allocation fraction, φ*, to warming for three scenarios of temperature dependence. Response without optimization (dashed curves) and with optimization (solid curves) are plotted as a function of the increase in temperature, up to +5°C. *Blue curves*, initial temperature *T*_0_ = 5°C. *Black curves*, *T*_0_ = *T*_ref_ = 20°C. *Red curves*, *T*_0_ = 30°C. (a) Baseline “kinetics only” scenario of temperature dependence. (b) Temperature‐dependent microbial turnover, with *E*_dM_ = 25 < *E*_v_^U^. (c) Temperature‐dependent microbial turnover, with *E*_dM_ = 55 > *E*_v_^U^. (d) Temperature‐dependent microbial growth efficiency (*γ*_*M*_), with *m* = −0.014. Parameters are set to their default values (table S1), except *I* = 5 10^−3^, *v*_0_^U^ = 10^5^, *E*_v_^U^ = 38, *c*_0_ = 1.17.
**Figure S9.** Global projections of soil C stock from 2010 to 2100 with litter input spatial and temporal variation. Litter input predictions are from the RCP8.5 scenario with the CCSM4 model.
**Figure S10.** Global projections of soil C stock from 2010 to 2100 with spatial variation in enzyme kinetics. Enzyme decomposition kinetics come from German et al. (2012)’s five sites of measurements. Global locations have been clustered in five groups based on biome characteristics and given each one of the five degrading enzyme types. Enzyme parameter values are in Table S3.
**Figure S11.** Global projections of soil C stock from 2010 to 2100 with spatial variation in enzyme kinetics and spatial and temporal variability in litter input. Enzyme decomposition kinetics come from German et al. (2012)’s five sites of measurements. Global locations have been clustered in five groups based on biome characteristics and given each one of the five degrading enzyme types. Enzyme parameter values are in Table S3. Litter input predictions are from the RCP8.5 scenario with the CCSM4 model.
**Figure S12.** Relative abundances of enzyme function classes (EFC) for (a) lignin and (b) carbohydrate degradation as a function of temperature, based on metagenomic data sampled in 200 sites across North America (Fan et al., in review). EFC is the ratio of enzyme‐degradative genes to the total gene count. The data are clustered into three temperature ranges: [−10, 2], [2, 10], and [10, 25]. These data reflect the potential effect of temperature as well as other environmental variables (e.g., moisture, litter quality).
**Figure S13.** Effect of temperature on optimal exoenzyme allocation: confronting eco‐evolutionary predictions with empirical data. In all panels, the green line represents the eco‐evolutionary model prediction for the optimal fraction of biomass allocated to enzyme production instead of biomass growth, φ*, as a function of temperature, *T*; the blue line estimations from the data and the blue area the 95% confidence interval (invisible in the bottom panels because very small). The relative change to the mean is calculated for both data and the model as (value‐mean)/mean, with the mean corresponding to the value at the average temperature. (a–c) Data from two‐month incubation experiments for three enzymes acting on (a) N‐acetyl‐β‐D‐glucosaminide (NAG), (b) β‐D‐glucopyranoside (BG), and (c) phosphate (PHOS). The average incubation temperature is 14.5°C. (d–f) Variation in Enzyme Function Classes (EFC) with temperature, estimated from metagenomes sampled in 200 sites across North America. A machine learning model was built to infer the EFC response to temperature only of (d) all carbon degrading enzymes, (e) carbohydrate degrading enzymes, and (f) lignin degrading enzymes. The gray bars represent the distribution of measurements (frequency) across temperature.
**Figure S14.** Experimental design of the enzyme assays conducted in 2013–2014.
**Figure S15.** Biomass‐specific enzyme activity as a function of assay temperature for four incubation temperatures. Dark blue: 4°C, light blue: 11°C, light red: 18°C, dark red: 25°C. (a) NAG, (b) BG, (c) PHOS. The points indicate the data, the lines represent the best fitted Arrhenius, and the shaded areas show their 95% confidence intervals. The dashed black line corresponds to the sensitivity to temperature of biomass‐specific enzyme activity with the values used in the theoretical model without evolution (Table S1).
**Table S1.** Parameter values.
**Table S2.** Sensitivity analysis of the nontrivial steady states of the microbe‐enzyme model to model parameters. The sensitivity to nonfraction parameters is tested over a range of zero to 100 and fractions are tested over 0 to 1, or to the range of existence and stability of the nontrivial steady state.
**Table S3.** Enzyme kinetics Arrhenius parameters from the five sites studied in German et al. (2012) used for the global projections with spatial variability in enzyme sensitivity to temperature (Figure S10).
**Table S4.** Literature review for the qualitative validation of our eco‐evolutionary model. Our model assumes a constant Q10, emphasizes eco‐evolutionary adaptation, and predicts that warming selects for soil microbes that allocate more resources to carbon degrading enzymes. The last column (“Model agreement and confidence”) indicates whether a study supports (+) or contradicts (−) our model predictions, with confidence levels denoted by +++ (high confidence) or −−− (high confidence in contradiction).

## Data Availability

The data and R scripts that support the findings of this study are openly available in Zenodo at https://doi.org/10.5281/zenodo.13940177. Soil temperature and litter data were obtained from NSF NCAR at (CCSM4).
