# Supplementary material for: Microbiome Adaptation Could Amplify Modeled Projections of Global Soil Carbon Loss With Climate Warming
Source: Glob Chang Biol. 2025 Jun 19;31(6):e70301. doi: 10.1111/gcb.70301 (PMC12179494; doi:10.1111/gcb.70301)
Supplement: Supplementary file 1 — Figure S1. Global distribution of soil surface temperature in 2010. Data are taken from the RCP8.5 scenario with the CCSM4 model (note: these do not account for the eco‐evolutionary carbon cycle feedbacks, reported here, that could further affect warming). Map lines delineate study areas and do not necessarily depict accepted national boundaries. Figure S2. Global distribution of soil carbon predictions in 2010. Soil carbon is the sum of the equilibrium of SOC and microbial biomass calculated with the AWB model with explicit trade‐off and optimization of allocation to enzymes to 2010 local soil temperature from the RCP8.5 scenario with the CCSM4 model. Parameter values are in Table S1. Map lines delineate study areas and do not necessarily depict accepted national boundaries. Figure S3. Earth terrestrial ecoregions (a) clustered into five biomes (b) for which enzyme kinetics parameters have been measured. The five biomes, and their reference location for which enzyme kinetics were measured, are tropical forest (Costa Rica, biome 1), temperate grassland (California, biome 2), temperate deciduous forest (West Virginia, biome 3), temperate/cold coniferous forest (Maine, biome 4), and boreal forest/tundra (Alaska, biome 5). Enzyme Arrhenius parameters are in Table S3. Map lines delineate study areas and do not necessarily depict accepted national boundaries. Figure S4. Projections of global CO2 flux from 2010 to 2100. (left y axis) Temporal change in global total CO2 flux without (dashed lines, blue shade) and with (solid lines, tan shade) microbial eco‐evolutionary optimization for three values of the competitive advantage model parameter (c 0 ); (right y axis, red) Temporal change in global average surface soil temperature between 2010 and 2100 from the RCP8.5 scenario with the CCSM4 model. Global CO2 flux is calculated every decade between 2010 and 2100, as the sum of local CO2 flux across all sites. Parameter values (e.g., c 0 ) are given in Table S1. Figure S5. Mod [file GCB-31-e70301-s001.docx]

Supplementary Materials for

**Microbiome adaptation could amplify global soil carbon loss**

**with climate warming**

Elsa Abs*, Scott R. Saleska, Steven D. Allison, Philippe Ciais, Yang Song, Michael N. Weintraub, Regis Ferriere

*Corresponding author: elsa.abs@lsce.ipsl.fr

**This PDF file includes:**

Notes S1 to S4

Figures S1 to S15

Tables S1 to S4

**Note S1. Expression of the steady states.**

At any given temperature *T*, the ecological model possesses either one globally stable equilibrium, or three equilibria (one of which is always unstable), depending on the enzyme allocation fraction *φ* (Fig. S3). A single (globally stable) equilibrium exists for *φ* < *φ*_min_ or *φ* > *φ*_max_, and is given by *M* = 0, *Z* = 0, *C* = *I*/*e*_C_, *D* = 0. In this case, microbial biomass is extinct and no decomposition occurs.

For *φ*_min_ < *φ* < *φ*_max_, the system may either converge to this no-decomposition equilibrium or stabilize at (or near) an alternative equilibrium where microbial biomass and enzyme production persist (called “non-trivial equilibrium”). This biologically active steady state can be solved for analytically:

$C=\frac{1}{e_{C}}\frac{\alpha-\sqrt{\alpha^{2}-4\beta}}{2\mu}$ $D=\frac{d_{M}K_{m}^{U}}{\lambda}$ $M=\frac{\gamma_{M}(1-\varphi)}{d_{M}\delta}\frac{\left( \alpha-\Delta_{M}+\sqrt{\alpha^{2}-4\beta} \right)}{2\mu}$ $Z=\frac{\gamma_{Z}\varphi}{d_{Z}\delta}\frac{\left( \alpha+\Delta_{Z}+\sqrt{\alpha^{2}-4\beta} \right)}{2\mu}$

where

$$\alpha=\mu\left( I-e_{C}K_{m}^{D} \right)+\varphi{\gamma_{Z}v}_{max}^{D}\rho$$

$$\beta=\mu\lambda\delta d_{Z}Ie_{C}K_{m}^{D}$$

$$\mu=\left( \varphi\gamma_{Z}v_{max}^{D}-d_{Z}\delta\right)\lambda$$

$$\Delta_{M}=2d_{Z}\delta\rho$$

$$\Delta_{Z}=-2d_{Z}\delta\rho$$

$$\lambda=\left( 1-\varphi\right)\gamma_{M}v_{max}^{U}-d_{M}$$

$$\delta=1-\left( 1-\varphi\right)\gamma_{M}-\varphi\gamma_{Z}$$

${\rho=e}_{C}K_{m}^{D}\lambda-e_{D}K_{m}^{U}d_{M}$

Although an analytical study of the stability of this equilibrium is out of reach, numerically we observed stability for most parameter values, based on the calculation of the Jacobian eigenvalues. Only for *φ* very close to *φ*_min_ we detected some cases of equilibrium instability, in which case the system converges toward a small limit cycle. *φ*_min_ therefore provides a good approximation for the lower bound of the range of *φ* over which the system persists. Boundary values, *φ*_min_ and *φ*_max_, are found where the unstable non-trivial equilibrium and the stable non-trivial equilibrium collide, *i.e.* when $\alpha^{2}-4\beta=0$, that we solved numerically.

**Note S2. Equations for the alternative scenarios of microbial temperature sensitivity.**

Two other scenarios have been proposed for the influence of temperature on decomposition. In the microbial growth efficiency (MGE) scenario, MGE (the fraction of carbon allocated to growth that actually contributes to microbial biomass, as opposed to being released as CO_2_ via growth respiration) decreases with warming [(Allison et al., 2010; Wieder et al., 2013)](https://paperpile.com/c/NeLSSp/kYR1P+g4sKW), which could be due to higher maintenance costs at higher temperature [(Sinsabaugh et al., 2013)](https://paperpile.com/c/NeLSSp/Xd5QP):

$$\gamma_{M}\left( T \right)= \gamma_{M,ref}-m\left( T-T_{ref} \right)$$

In the microbial mortality scenario, the microbial death rate also increases with temperature:

$$d_{M}\left( T \right)= d_{M0}e^{-\frac{E_{dM}}{R\left( T+273 \right)}}$$

This could be due to a higher risk of predation or pathogenic infection at higher temperatures, or faster microbial senescence due to higher protein turnover [(Hagerty et al., 2014)](https://paperpile.com/c/NeLSSp/fO2LR).

**Note S3. Latitudinal data on microbial metagenomics.**

We analyzed latitudinal data on microbial metagenomics from 575 samples collected across 42 sites, spanning 15 of 17 NEON eco-regions in North America (Fan et al., in review). The model response to temperature change is evaluated across spatial gradients, using a space for time substitution hypothesis, which is a very strong assumption. As a proxy for enzyme allocation to carbon degradation, we used the ratio of carbon-degrading genes to total gene count, referred to as the relative abundance of Enzyme Function Classes (EFC). Consistent with our eco-evolutionary model’s predictions, we observed a non-linear increase in lignin EFC with temperature (Fig. S12a). In contrast, carbohydrate EFC showed no discernible pattern (Fig. S12b). Focusing on lignin is reasonable, as it serves as a proxy for slow-degrading SOC, given its long decomposition timescales compared to carbohydrates, making it particularly relevant to climate change timescales. However, the sampled sites varied in environmental conditions beyond temperature (e.g., precipitation, litter input). When Fan et al. (in review) used an Accumulated Local Effects (ALE) analysis to isolate the effect of temperature, the observed patterns showed a poor fit with our model predictions (Fig. S13d-f).

**Note S4. Experimental incubations and enzyme assays across temperature.**

To complement the insights from latitudinal samplings reflecting evolutionary outcomes over millions of years, we also compared our eco-evolutionary model’s predictions with exoenzyme activity data from soil samples exposed to temperature warming during incubation experiments. These experiments are of short duration (two months at different temperatures) and we make the strong assumption that our modeled long term response of *φ* to warming over the next century can be compared to these short term empirical data. The field experiment from which these samples were collected was reported in [(McCulley et al., 2014)](https://paperpile.com/c/NeLSSp/zsGMR). In the winter of 2013-2014, soil samples were taken from 6 plots, 3 replicates from ambient plots and 3 replicates from soil heated for 4 years (+3°C day and night). The 6 samples were assayed for 4 hydrolytic enzymes (4-N-acetyl-β-glucosaminidase (NAG), 4-β-D-glucosidase (BG), L-leucine-amino-peptidase (LAP) and phosphomonoesterase (PHOS)) and 2 oxidative enzymes (Phenol oxidase (PHENOX), Peroxidase (PEROX)) at 4 °C, 11 °C, 18 °C and 25 °C. The 6 samples were then incubated for 2 months at these same 4 temperatures, and then assayed again at these same 4 temperatures, leading to 192 assays (Fig. S14). The enzyme assay methods are detailed in [(Slaughter et al., 2015)](https://paperpile.com/c/NeLSSp/XqSx7). For our analysis, the effect of plot temperature was negligible, so we combined data from control and heated plots and focused on the effect of incubation temperature.

The activities of the enzymes assayed in this study follow Michaelis-Menten kinetics, and therefore saturate with substrate concentration and scale with enzyme concentration. Their maximal rates obey the Arrhenius equation with respect to temperature. Thus, an enzyme’s activity is given by:

$$Enzyme activity =\left[ E \right]\frac{V_{max}(T) \left[ S \right]}{K_{m}+\left[ S \right]}=\left[ E \right]\frac{Ae^{\frac{-Ea}{RT}}\left[ S \right]}{K_{m}+\left[ S \right]}$$

where [*S*] and [*E*] are the substrate and enzyme concentrations, *V_max_* and *K_m_* are the maximum activity rate and the half-saturation constant of the Michaelis-Menten equation, *A* is the Arrhenius pre-exponential constant, *E_a_* the activation energy, and *R* the gas constant. In treatments with added substrate, where [*S*] >> *K_m_*, enzyme activity simplifies to:

$$Enzyme activity=\left[ E \right]Ae^{\frac{-Ea}{RT}}$$

The logarithm of enzyme activity is a linear function of the reciprocal of the assay temperature, where the intercept equals the logarithm of $\left[ E \right]A$ and the slope equals $\frac{-Ea}{R}$. We plotted this function for each of the four incubation temperatures for each enzyme to examine evidence for adaptation to the temperature during the 2-month incubation period (Fig. S15). We replaced the negative values of enzyme activity, which generally indicate very low activity, with 0. We also took the treatment with added substrate (+L) to avoid substrate limitation effects. Then, we assumed biomass-specific enzyme activity to be an Arrhenius function of assay temperature and identify the best Arrhenius fit for each incubation temperature.

Differences in slope (i.e., in activation energy, *E_a_*) would indicate an adaptation in the enzymes' temperature sensitivity, while differences in the intercept (i.e., in $\left[ E \right]A$) would suggest adaptations—likely ecological at this timescale—in enzyme concentration (*[E]*), kinetic capacity (*A*), or both. To control for differences in community size, we used biomass-specific enzyme activity. Among the six enzymes tested, the warm-adapted N-acetyl-β-glucosaminidase (NAG) showed a higher intercept, supporting our model's prediction that microbes invest more in enzyme production in warmer regions (Fig. S15a). However, this pattern was not observed for β-glucosidase (BG) and phosphatase (PHOS) (Fig. S15b, c). This discrepancy could suggest three possibilities: our model might be incorrect, the concentration of cold-adapted enzymes could be lower while their kinetic capacity (A) is significantly higher, or cold-adapted enzyme turnover ($r_{loss}$) is even lower than enzyme production ($E_{production}$), leading to higher enzyme concentration ($\left[ E \right]$):

$\frac{d\left[ E \right]}{dt}=E_{production}(T)-r_{loss}(T)\left[ E \right]$.

In Fig. S13a-c, we aimed to compare the estimated markers of adaptation from the data with the predictions from the eco-evolutionary model. The closest estimate to enzyme allocation we can derive from the data is [*E*]*A*, where enzyme concentration [*E*] is a product of enzyme allocation and the enzyme quantity produced per unit of allocated resource. We assumed that the kinetic capacity (*A*) and the enzyme quantity produced per unit of allocated resource do not vary between incubation temperatures in the experiment, so that any variation is due solely to changes in enzyme allocation. We compared the variation relative to the mean at the averaged incubation temperature (14.5 °C). To do this, we calculated the biomass-specific activity at 14.5 °C for each incubation temperature (Fig. S15), took the mean of these four values, and then calculated the difference (value - mean) normalized by the mean.

Of the six enzymes assayed, one (N-acetyl-β-D-glucosaminide or NAG) supported our model’s predictions, with the warm-incubated samples showing overall higher enzyme activity than the cold-incubated ones (Fig. 13a). However, this community-level activity is determined by both enzyme concentration and enzyme-level activity, and enzyme concentration itself depends on enzyme production and degradation. Therefore, we would need two additional measurements (enzyme concentration and production) to complete the validation of the exoenzyme allocation predictions from our eco-evolutionary model.


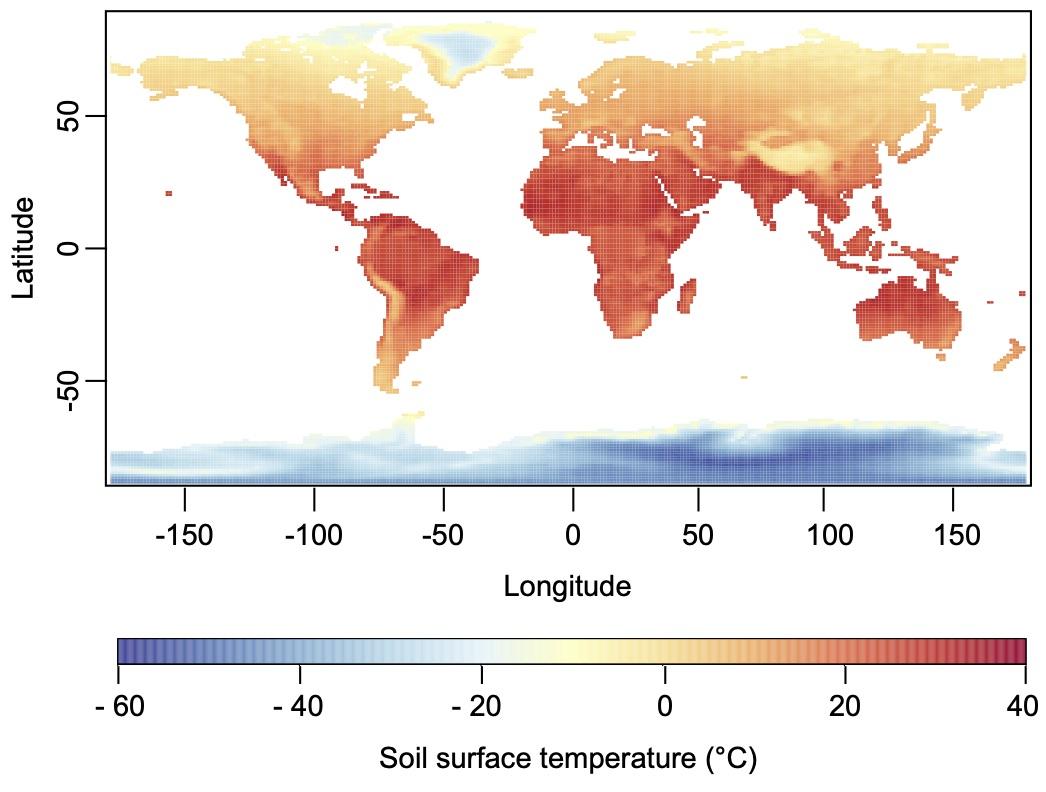


**Fig. S1. Global distribution of soil surface temperature in 2010.** Data are taken from the RCP8.5 scenario with the CCSM4 model (note: these do not account for the eco-evolutionary carbon cycle feedbacks, reported here, that could further affect warming). Map lines delineate study areas and do not necessarily depict accepted national boundaries.


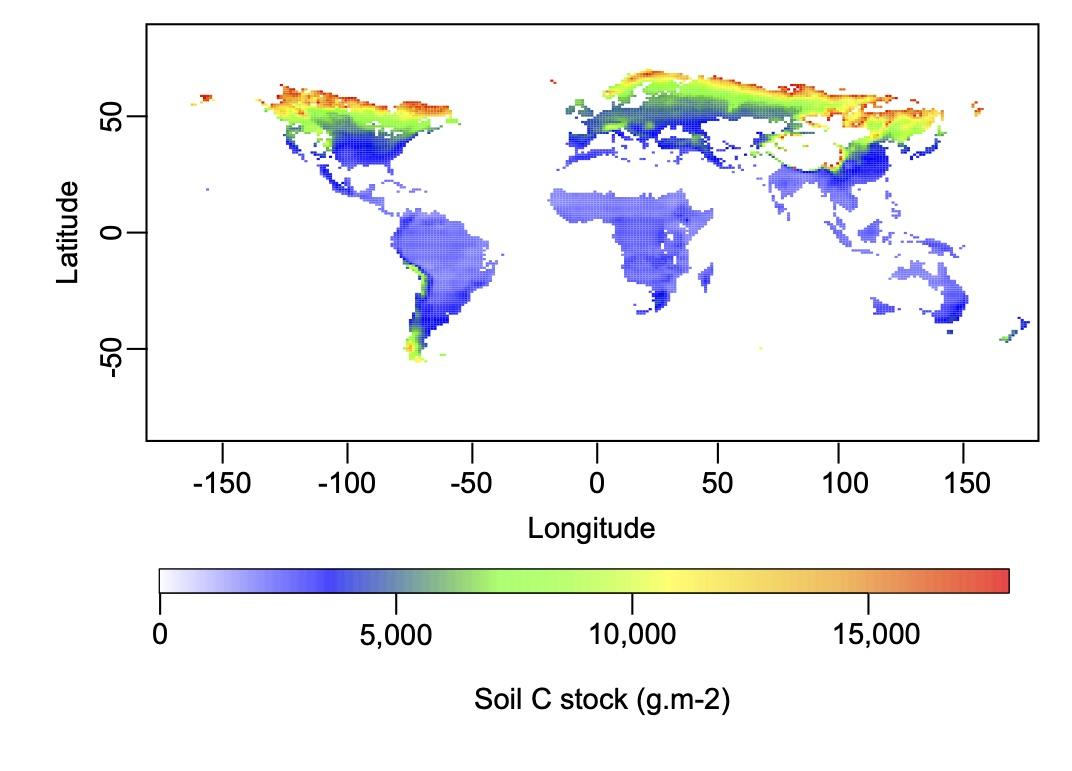


**Fig. S2. Global distribution of soil carbon predictions in 2010.** Soil carbon is the sum of the equilibrium of SOC and microbial biomass calculated with the AWB model with explicit trade-off and optimization of allocation to enzymes to 2010 local soil temperature from the RCP8.5 scenario with the CCSM4 model. Parameter values are in Table S1. Map lines delineate study areas and do not necessarily depict accepted national boundaries.

**
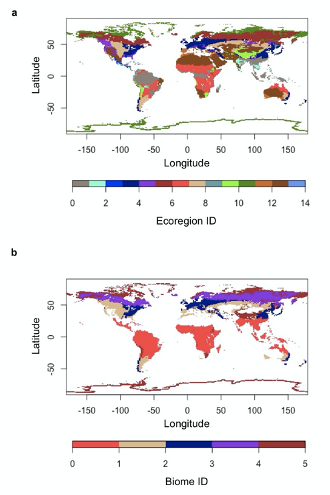
**

**Fig. S3. Earth terrestrial ecoregions (a) clustered into five biomes (b) for which enzyme kinetics parameters have been measured.** The five biomes, and their reference location for which enzyme kinetics were measured, are tropical forest (Costa Rica, biome 1), temperate grassland (California, biome 2), temperate deciduous forest (West Virginia, biome 3), temperate/cold coniferous forest (Maine, biome 4), and boreal forest/tundra (Alaska, biome 5). Enzyme Arrhenius parameters are in Table S3. Map lines delineate study areas and do not necessarily depict accepted national boundaries.


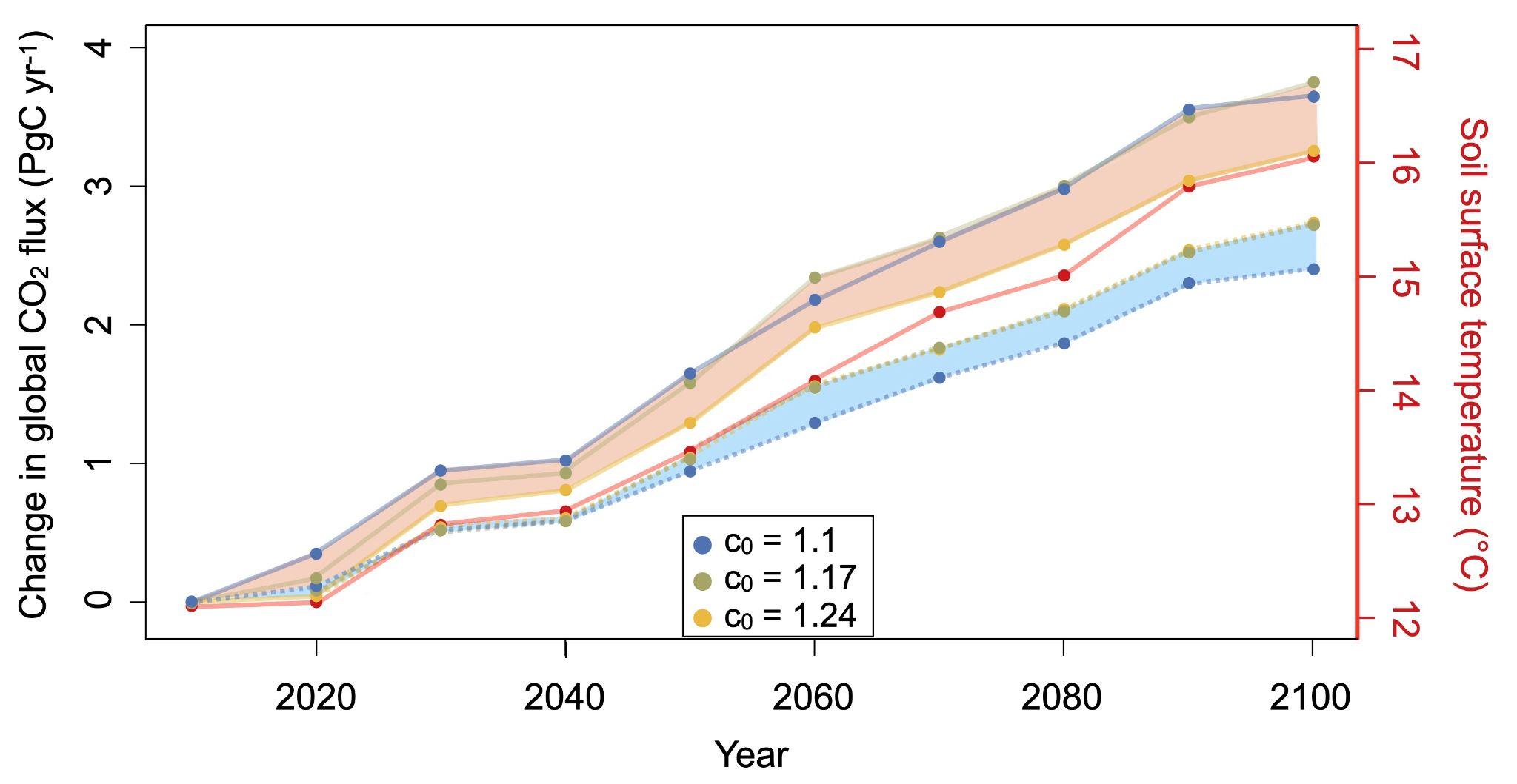


**Fig. S4. Projections of global CO_2_ flux from 2010 to 2100.** (left y axis) Temporal change in global total CO_2_ flux without (dashed lines, blue shade) and with (solid lines, tan shade) microbial eco-evolutionary optimization for three values of the competitive advantage model parameter (*c_0_*); (right y axis, red) Temporal change in global average surface soil temperature between 2010 and 2100 from the RCP8.5 scenario with the CCSM4 model. Global CO_2_ flux is calculated every decade between 2010 and 2100, as the sum of local CO_2_ flux across all sites. Parameter values (e.g., *c_0_*) are given in Table S1.

**
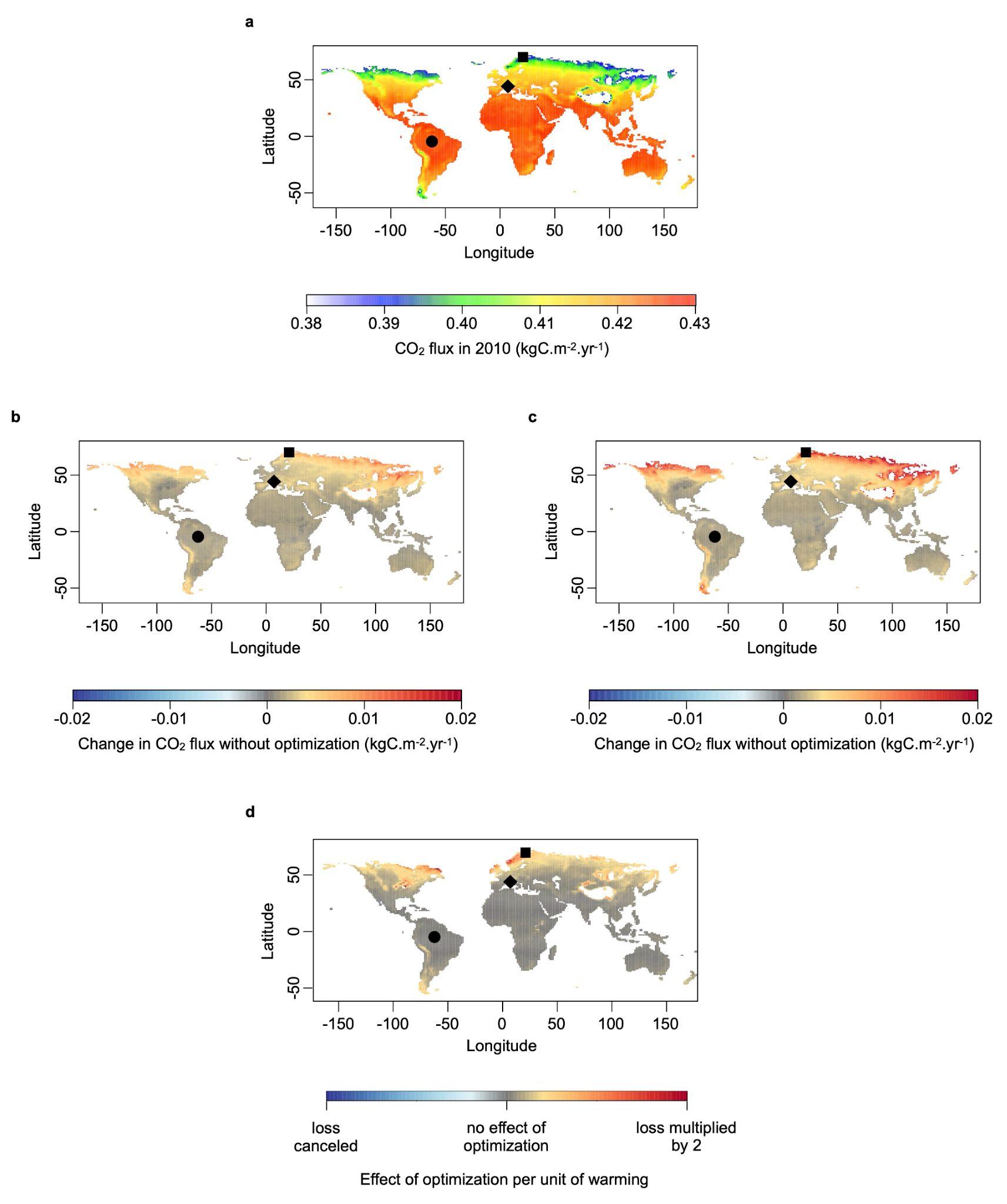
**

**Fig. S5. Model projections under the RCP8.5 soil temperature scenarios.** **(a)** Global distribution of CO_2_ flux predictions in 2010. **(b)** Changes in CO_2_ flux between 2010 and 2100 without optimization. **(c)** Changes in CO_2_ flux between 2010 and 2100 with optimization. **(d)** Effect of eco-evolutionary optimization per unit of warming between 2010 and 2100. The effect of optimization is the difference between the model runs with and without optimization. Map lines delineate study areas and do not necessarily depict accepted national boundaries.


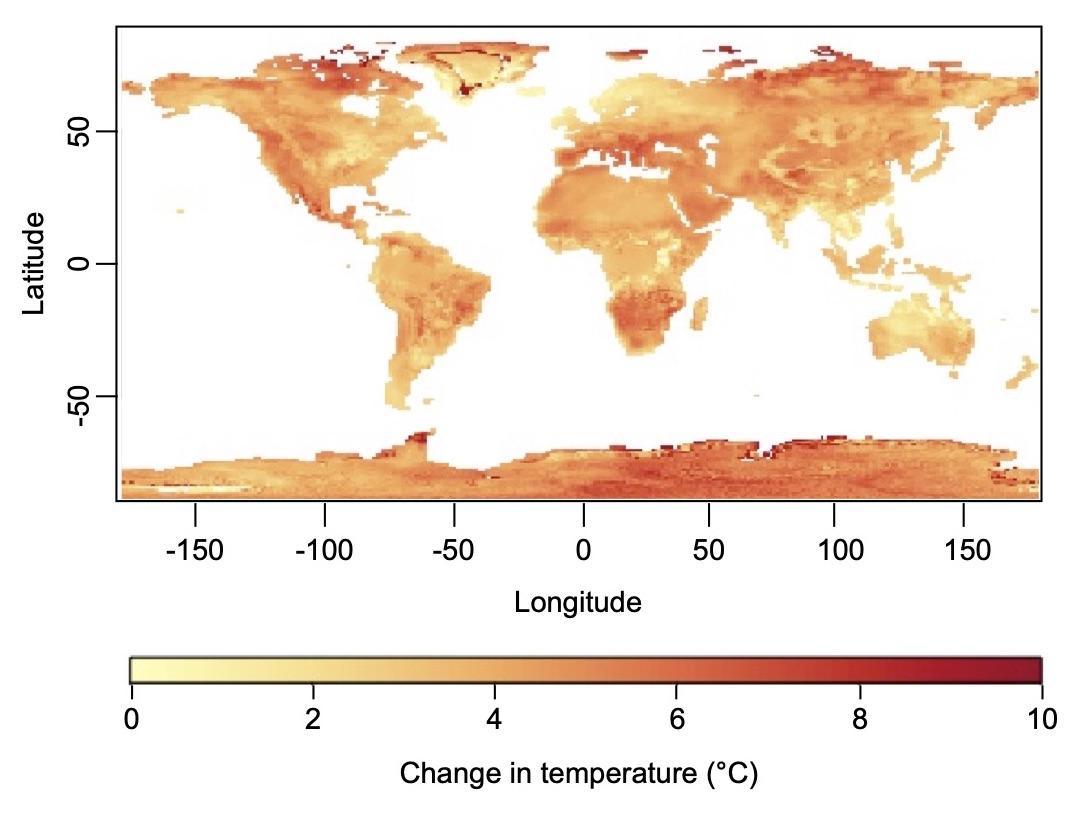


**Fig. S6. Global distribution of change in surface soil temperature from 2010 to 2100.** Predictions from the RCP8.5 scenario with the CCSM4 model. Map lines delineate study areas and do not necessarily depict accepted national boundaries.

**
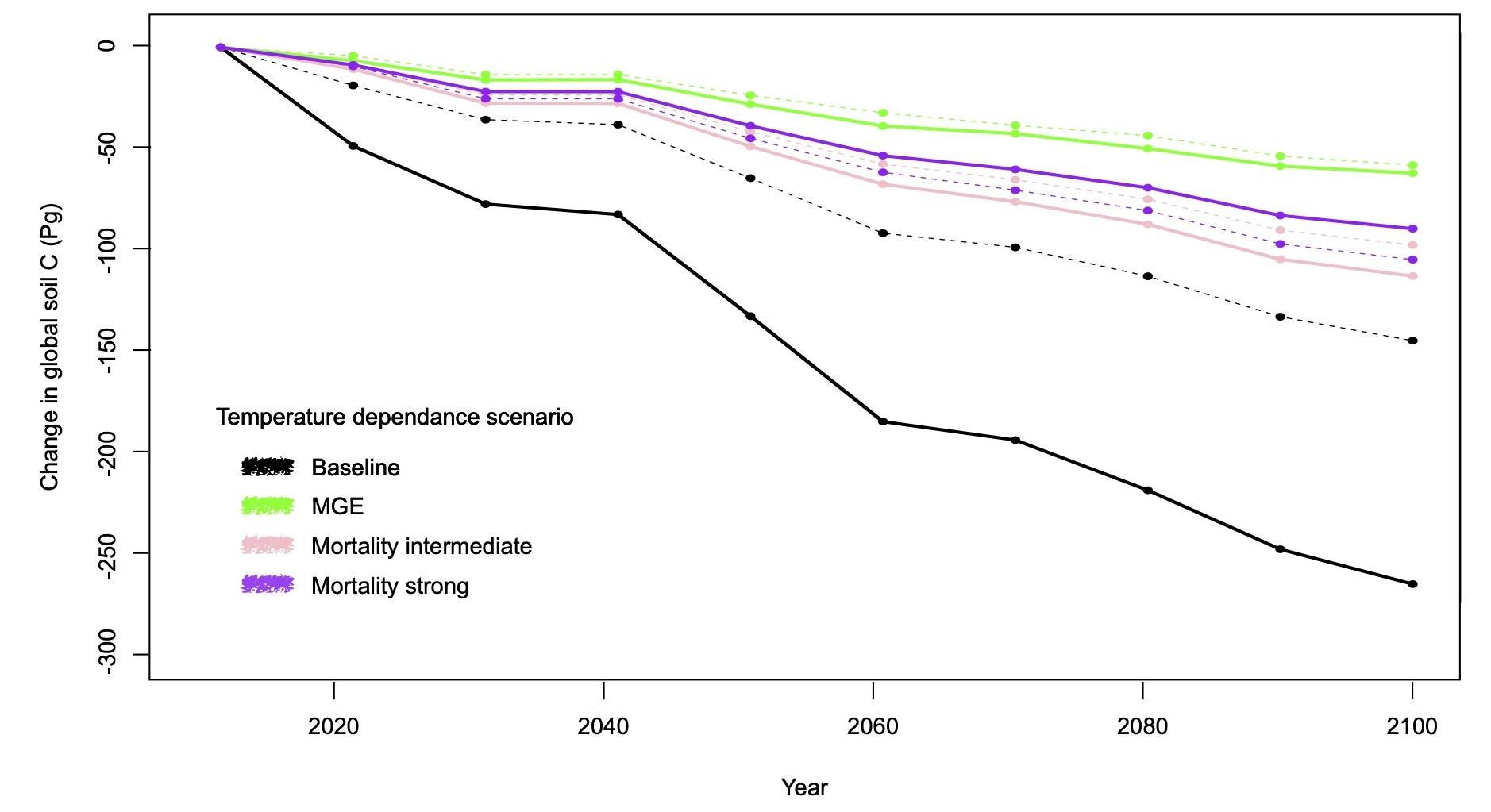
**

**Fig. S7. Global projections of soil C stock from 2010 to 2100 for reference eco-evolutionary optimization scenario (black) and for 3 other scenarios of microbial temperature sensitivity within the eco-evolutionary model:** (green) microbial growth efficiency decreases with warming, (pink) microbial mortality increases with warming, (purple) microbial mortality increases strongly with warming. Each color comes with 2 models’ predictions: (dashed) no optimization, (solid) with eco-evo optimization.


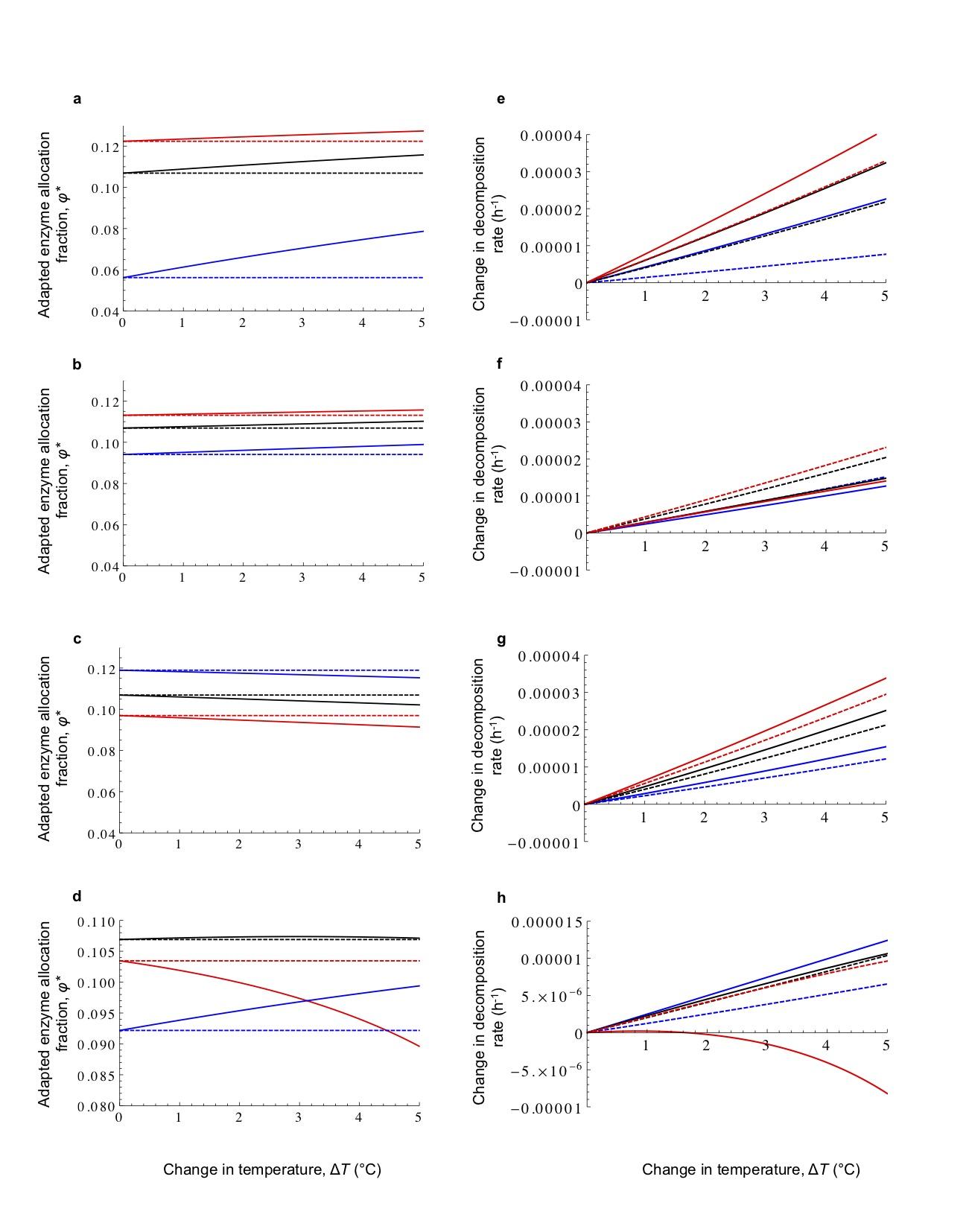


**Fig. S8. Responses of the enzyme allocation fraction, *φ**, to warming for three scenarios of temperature dependence.** Response without optimization (dashed curves) and with optimization (solid curves) are plotted as a function of the increase in temperature, up to + 5°C. *Blue curves*, initial temperature *T*_0_ = 5°C. *Black curves*, *T*_0_ = *T*_ref_ = 20°C. *Red curves*, *T*_0_ = 30°C. **(a)** Baseline ‘kinetics only’ scenario of temperature dependence. **(b)** Temperature-dependent microbial turnover, with *E*_dM_ = 25 < *E*_v_^U^. **(c)** Temperature-dependent microbial turnover, with *E*_dM_ = 55 > *E*_v_^U^. **(d)** Temperature-dependent microbial growth efficiency (*γ_M_*), with *m* = - 0.014. Parameters are set to their default values (table S1), except *I* = 5 10^-3^, *v*_0_^U^ = 10^5^, *E*_v_^U^ = 38, *c*_0_ = 1.17.

**
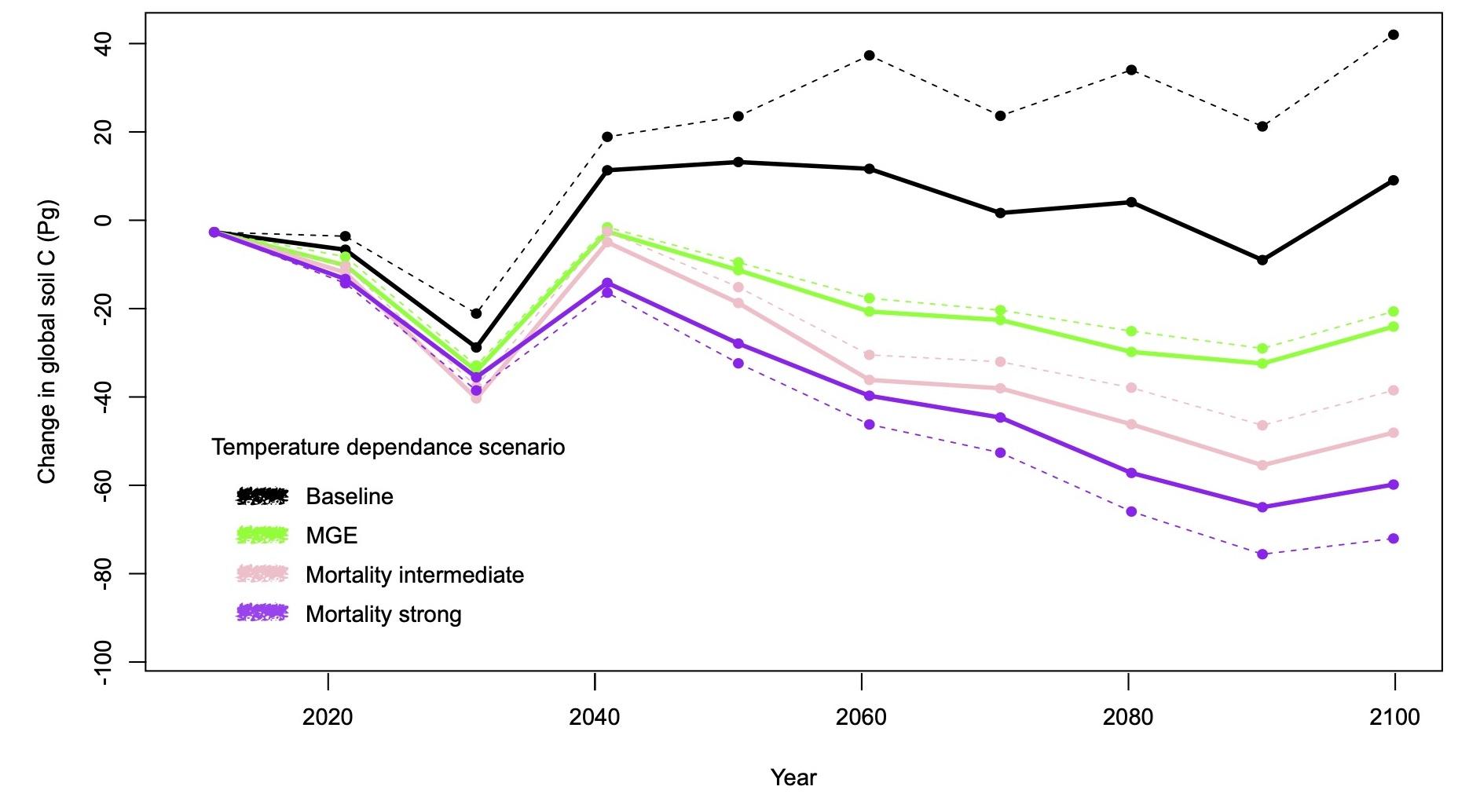
**

**Fig. S9. Global projections of soil C stock from 2010 to 2100 with litter input spatial and temporal variation.** Litter input predictions are from the RCP8.5 scenario with the CCSM4 model.

**
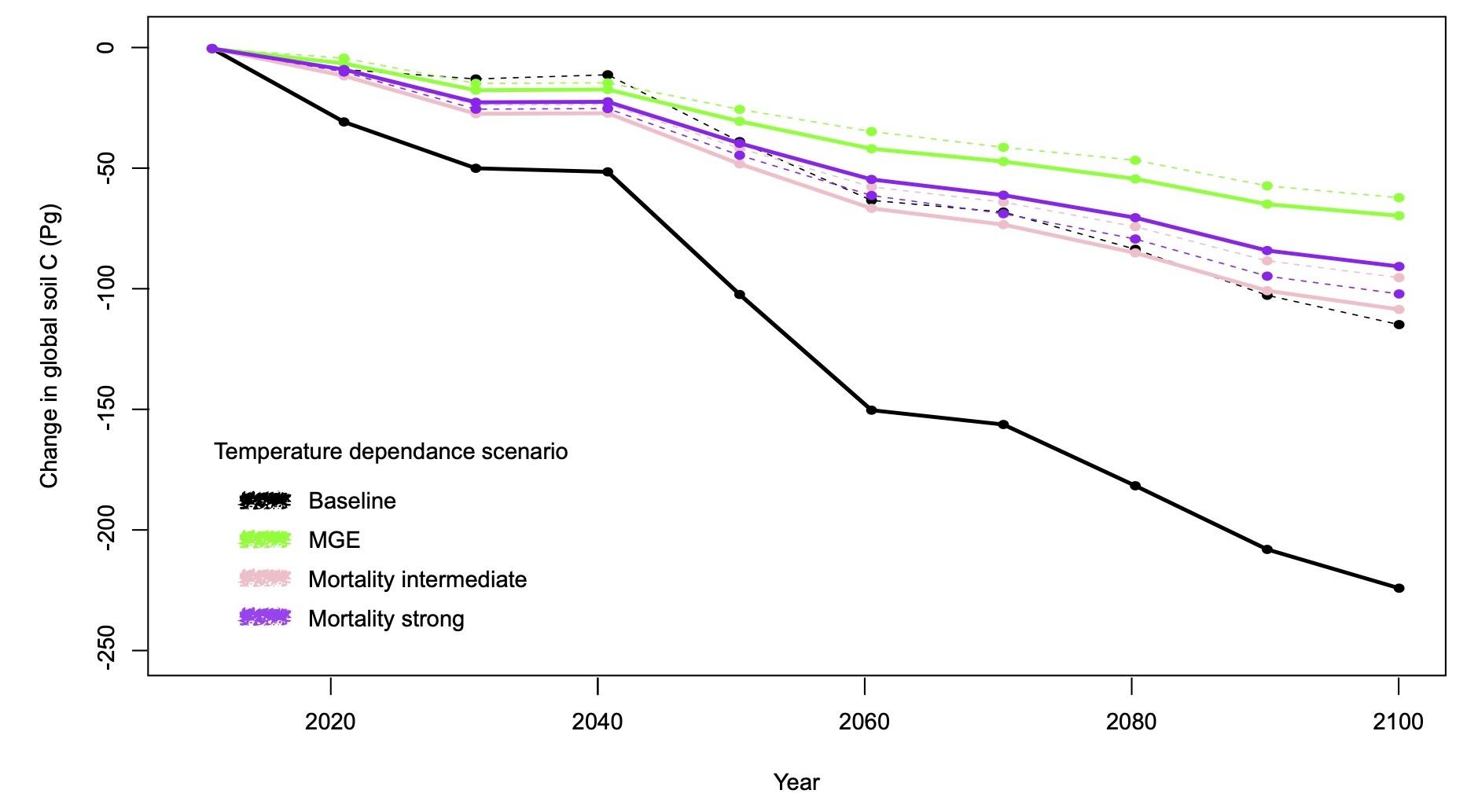
**

**Fig. S10. Global projections of soil C stock from 2010 to 2100 with spatial variation in enzyme kinetics.** Enzyme decomposition kinetics come from German et al. [(2012)](https://paperpile.com/c/NeLSSp/Q1hNH)’s five sites of measurements. Global locations have been clustered in five groups based on biome characteristics and given each one of the 5 degrading enzyme types. Enzyme parameter values are in Table S3.

**
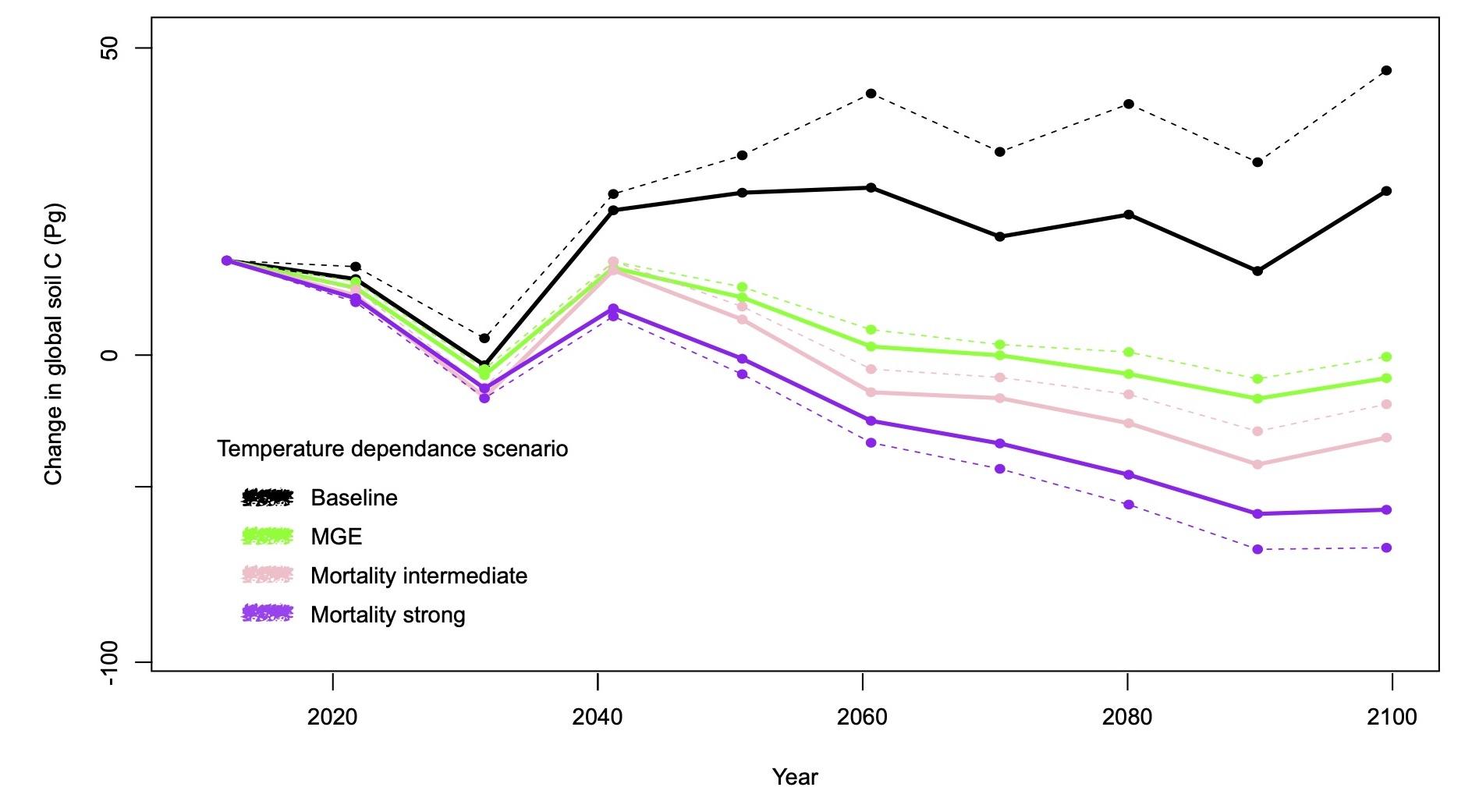
**

**Fig. S11. Global projections of soil C stock from 2010 to 2100 with spatial variation in enzyme kinetics and spatial and temporal variability in litter input.** Enzyme decomposition kinetics come from German et al. (2012)’s five sites of measurements. Global locations have been clustered in five groups based on biome characteristics and given each one of the 5 degrading enzyme types. Enzyme parameter values are in Table S3. Litter input predictions are from the RCP8.5 scenario with the CCSM4 model.


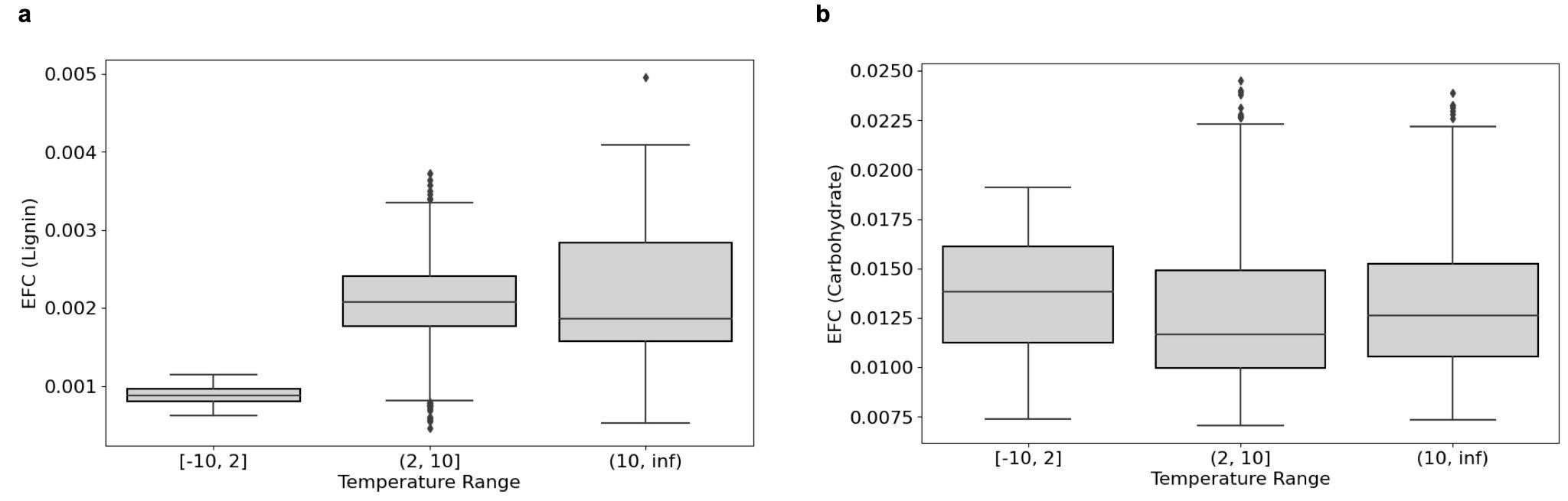


**Fig. S12. Relative abundances of Enzyme Function Classes (EFC) for (a) lignin and (b) carbohydrate degradation as a function of temperature**, based on metagenomic data sampled in 200 sites across North America (Fan et al., in review). EFC is the ratio of enzyme-degradative genes to the total gene count. The data are clustered into three temperature ranges: [-10,2], [2,10], [10,25]. These data reflect the potential effect of temperature as well as other environmental variables (e.g., moisture, litter quality).

**
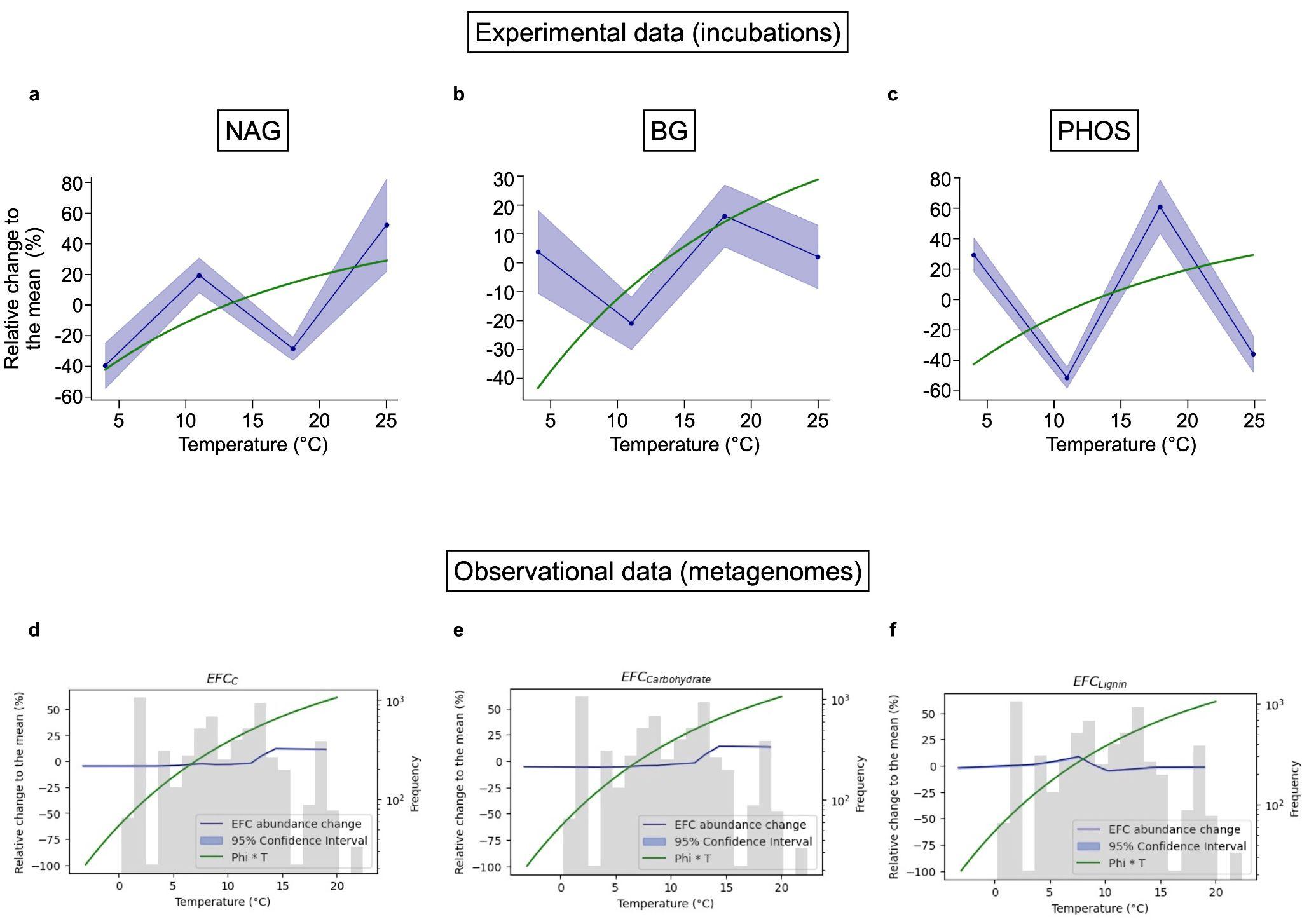
**

**Fig. S13. Effect of temperature on optimal exoenzyme allocation: confronting eco-evolutionary predictions with empirical data.** In all panels, the green line represents the eco-evolutionary model prediction for the optimal fraction of biomass allocated to enzyme production instead of biomass growth, φ*, as a function of temperature, *T*; the blue line estimations from the data and the blue area the 95% confidence interval (invisible in the bottom panels because very small). The relative change to the mean is calculated for both data and the model as (value-mean)/mean, with the mean corresponding to the value at the average temperature. **(a-c)** Data from two-months incubation experiments for three enzymes acting on **(a)** N-acetyl-β-D-glucosaminide (NAG), **(b)** β-D-glucopyranoside (BG), and **(c)** phosphate (PHOS). The average incubation temperature is 14.5°C. **(d-f)** Variation in Enzyme Function Classes (EFC) with temperature, estimated from metagenomes sampled in 200 sites across North America. A machine learning model was built to infer the EFC response to temperature only of **(d)** all carbon degrading enzymes, **(e)** carbohydrate degrading enzymes, and **(f)** lignin degrading enzymes. The gray bars represent the distribution of measurements (frequency) across temperature.


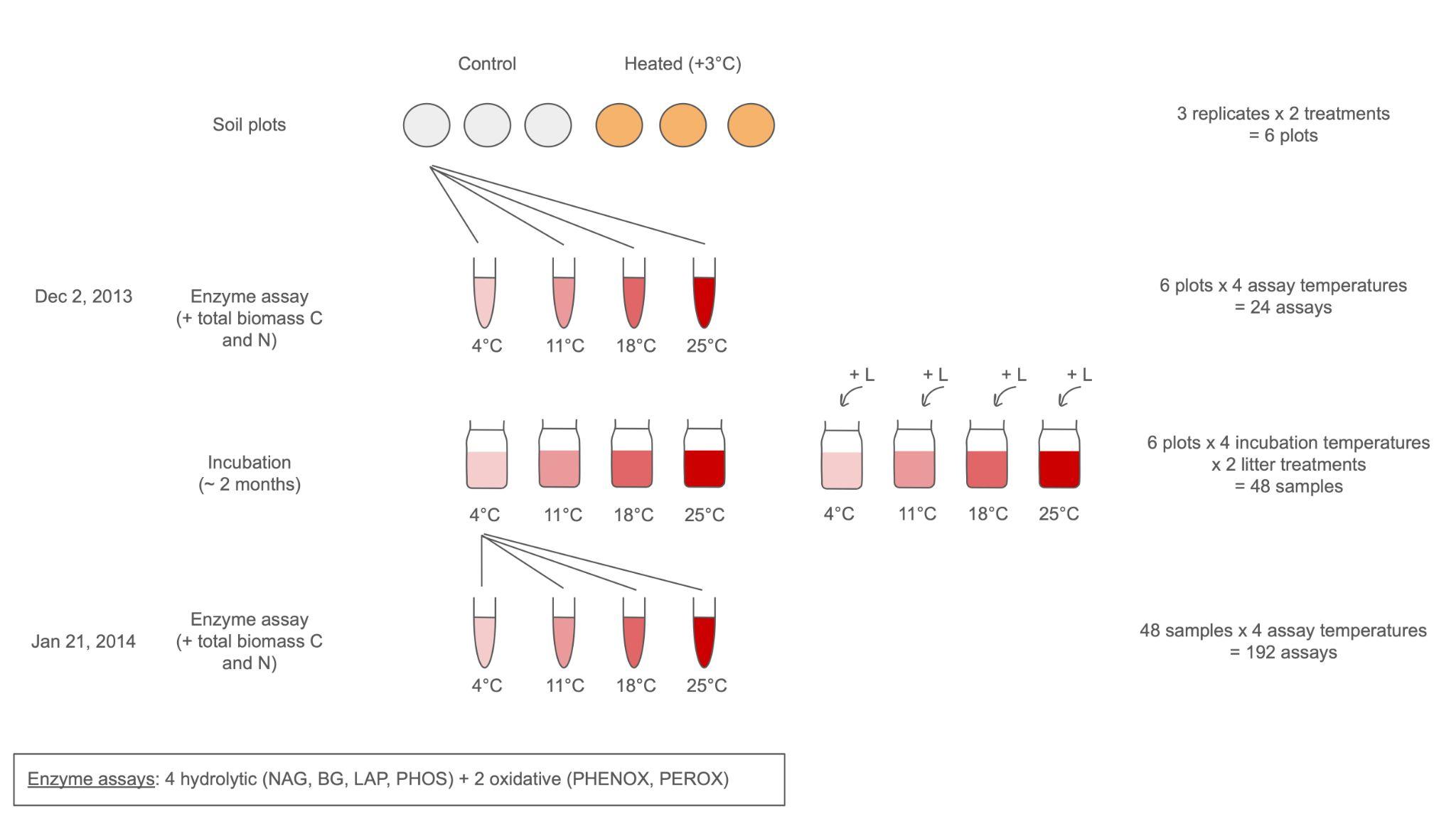


**Fig. S14. Experimental design of the enzyme assays** conducted in 2013-2014.


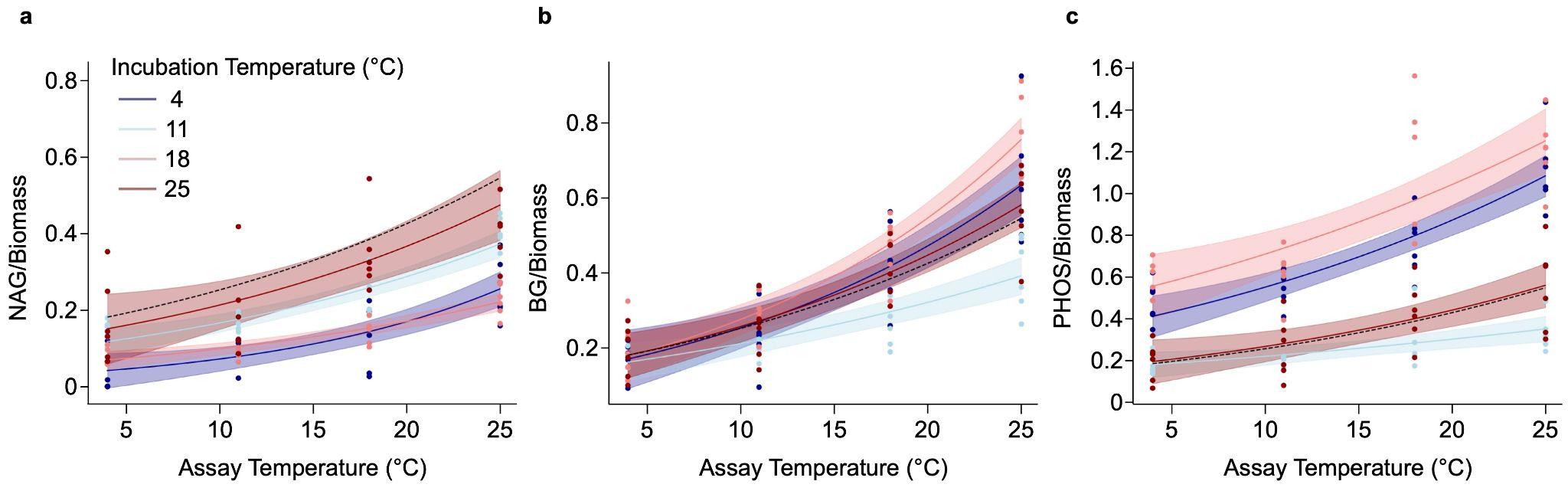


**Fig. S15. Biomass-specific enzyme activity** as a function of assay temperature for four incubation temperatures. Dark blue: 4°C, light blue: 11°C, light red: 18°C, dark red: 25°C. (a) NAG, (b) BG, (c) PHOS. The points indicate the data, the lines represent the best fitted Arrhenius, and the shaded areas show their 95% confidence intervals. The dashed black line corresponds to the sensitivity to temperature of biomass-specific enzyme activity with the values used in the theoretical model without evolution (Table S1).

**Table S1. Parameter values.**

| Parameter | Unit | Description | Default value |
| --- | --- | --- | --- |
| *T*_0_, *T*_ref_ | °C | initial temperature | 20 |
| *φ* |  | enzyme allocation fraction | 0.1 |
| *γ*_M_, *γ*_M,ref_ |  | microbial growth efficiency | 0.31 |
| *γ*_Z_ |  | enzyme production efficiency | 0.4 |
| *d*_M_, *d*_M,ref_ | h^-1^ | microbial mortality rate | 2 10^-4^ |
| *d*_Z_ | h^-1^ | enzyme deactivation rate | 2 10^-3^ |
| $v_{0}^{U}$ | mg D cm^-3^ (mg M cm^-3^)^-1^ h^-1^ | Arrhenius coefficient of uptake rate | 10^5^ |
| $v_{0}^{D}$ | mg C cm^-3^ (mg Z cm^-3^)^-1^ h^-1^ | Arrhenius coefficient of decomposition rate | 1.15 10^6^ |
| $K_{0}^{U}$ | mg D cm^-3^ | Arrhenius coefficient of uptake half-saturation constant | 1.6 10^3^ |
| $K_{0}^{D}$ | mg C cm^-3^ | Arrhenius coefficient of decomposition half-saturation constant | 3.3 10^4^ |
| $E_{v}^{U}$ | kJ mol^-1^ | activation energy of uptake rate | 38 |
| $E_{v}^{D}$ | kJ mol^-1^ | activation energy of decomposition rate | 36.1 |
| $E_{K}^{U}$ | kJ mol^-1^ | activation energy of uptake half-saturation constant | 21 |
| $E_{K}^{D}$ | kJ mol^-1^ | activation energy of decomposition half-saturation constant | 9.7 |
| *E*_dM_ | kJ mol^-1^ | activation energy of microbial turnover | 0, 25, 55 |
| *I* | mg C cm^-3^ h^-1^ | SOC input (litter) | 5 10^-3^ |
| *e*_C_ | h^-1^ | SOC leaching rate | 10^-6^ |
| *e*_D_ | h^-1^ | DOC leaching rate | 10^-2^ |
| *c*_0_ |  | local competitive advantage to stronger exoenzyme producers (competition asymmetry) | 1.17 |

**Table S2. Sensitivity analysis** of the non-trivial steady states of the microbe-enzyme model to model parameters. The sensitivity to non fraction parameters is tested over a range of zero to 100 and fractions are tested over 0 to 1, or to the range of existence and stability of the non trivial steady state.

| Parameter | Lower bound | Upper bound | Sensitivity (absolute value) | | | |
| --- | --- | --- | --- | --- | --- | --- |
|  |  |  | C | D | M | Z |
| *T* | 4.9 | 490 | 1.62 | 0.83 | 0.06 | 0.06 |
| *φ* | 0.19 | 0.95 | 0.97 | 1.75 | 1.75 | 0.98 |
| *γ*_M_ | 0.19 | 0.95 | 1.32 | 1 | 2.34 | 1.34 |
| *γ*_Z_ | 0.19 | 0.95 | 1.18 | 0 | 0.24 | 1.24 |
| *d*_M_ | 3.8 10^-6^ | 3.8 10^-4^ | 0.005 | 1 | 1.01 | 0.005 |
| *d*_Z_ | 4.6 10^-5^ | 4.6 10^-3^ | 1.05 | 0 | 0.07 | 1.07 |
| $v_{0}^{U}$ | 2.1 10^5^ | 2.1 10^7^ | 0.01 | 1 | 0.01 | 0.01 |
| $v_{0}^{D}$ | 5.1 10^5^ | 5.1 10^7^ | 1.05 | 0 | 0.07 | 0.07 |
| $K_{0}^{U}$ | 60 | 6000 | 0.01 | 1 | 0.01 | 0.01 |
| $K_{0}^{D}$ | 900 | 9 10^4^ | 1 | 0 | 0.08 | 0.08 |
| $E_{v}^{U}$ | 0.38 | 38 | 0.01 | 3.35 | 0.01 | 0.01 |
| $E_{v}^{D}$ | 0.38 | 38 | 3.4 | 0 | 0.07 | 0.07 |
| $E_{K}^{U}$ | 18 | 1800 | 0.01 | 159 | 0.01 | 0.01 |
| $E_{K}^{D}$ | 7.3 | 730 | 7.58 | 0 | 0.08 | 0.08 |
| *I* | 2.8 10^-4^ | 2.8 10^-2^ | 0.005 | 0 | 1.05 | 1.05 |
| *e*_C_ | 5 10^-8^ | 5 10^-6^ | 0.003 | 0 | 0.18 | 0.18 |
| *e*_D_ | 3.8 10^-4^ | 3.8 10^-2^ | 0.01 | 0 | 0.01 | 0.01 |
| *c*_0_ | 1.1 | 25 | 0.75 | 1 | 0.99 | 0.76 |

**Table S3. Enzyme kinetics Arrhenius parameters** from the five sites studied in German et al. (2012) used for the global projections with spatial variability in enzyme sensitivity to temperature (Fig. S10).

| Parameter | Location | | | | |
| --- | --- | --- | --- | --- | --- |
|  | Alaska | Maine | West Virginia | California | Costa Rica |
| *T_0_* (°C) | 0 | 5 | 9 | 17 | 26 |
| *v_0_^D^* (h^-1^) | 7.7 10^7^ | 7.73 10^9^ | 1.35 10^8^ | 1.15 10^6^ | 1.23 10^8^ |
| *E_v_^D^* (kJ mol^-1^) | 43.7 | 50.6 | 47.2 | 36.1 | 48.9 |
| *K_0_^D^* (mg cm^-3^) | 2.79 10^7^ | 2.1 10^7^ | 3.1 10^6^ | 3.3 10^4^ | 4.4 10^3^ |
| *E_K_^D^* (kJ mol^-1^) | 26.2 | 23.8 | 21.4 | 9.7 | 5.2 |

**Table S4. Literature review for the qualitative validation of our eco-evolutionary model.** Our model assumes a constant Q10, emphasizes eco-evolutionary adaptation, and predicts that warming selects for soil microbes that allocate more resources to carbon-degrading enzymes. The last column (“Model agreement and confidence”) indicates whether a study supports (+) or contradicts (-) our model predictions, with confidence levels denoted by +++ (high confidence) or --- (high confidence in contradiction).

| Article | Experimental setup | Analysis approach | Key findings about warmed samples | Strength for model validation | Limitations for model validation | Model agreement and confidence |
| --- | --- | --- | --- | --- | --- | --- |
| [DeAngelis et al., 2015](https://paperpile.com/c/NeLSSp/AKxc) | Harvard Forest (+5°C, 5–20 years, coarse-loamy soil) | Community composition analysis with 16S + qPCR | Community shift after 20 years in organic soil  Higher abundance of taxa associated with complex organic matter degradation  No shift at 5 and 8 years, or in the mineral soil | Shifts in community structure indicates eco-evolutionary adaptation, showing that the response is not solely due to plasticity | 16S may lack resolution to detect shifts in carbon-degrading capacity | ++ |
| [Pold et al., 2015](https://paperpile.com/c/NeLSSp/tJEE) | Same as DeAngelis 2015, but distinct sampling effort (23 years) | Enzyme assays  at single temperature | Higher potential lignin-degrading enzyme activity in the unamended beads, but lower in the lignin beads | Enzyme assays better approximate decay potential than respiration measurements | Cell count does not capture change in biomass, so biomass might have been underestimated | + |
|  |  |  |  |  |  |  |
|  |  | Taxonomic analysis | Community shift toward taxa associated with lignin degradation | Shifts in community structure indicates eco-evolutionary adaptation, showing that the response is not solely due to plasticity | Without genomic or functional analyses, it is unclear whether these changes reflect an actual increase in lignin-degrading potential |  |
| [Pold et al., 2016](https://paperpile.com/c/NeLSSp/sRMX) | Same as DeAngelis 2015, but distinct sampling effort | Taxonomic and phylogenetic analysis | Carbohydrate degradation traits are conserved across taxa, implying adaptation at the community level rather than plasticity | Potential functions are valuable because they represent capacities that could be expressed across a range of conditions instead of one or a few conditions only | Lacks metatranscriptomic analysis to link potential to realized functions | ++ |
|  |  | Degradation assays of isolates | Higher capacity to degrade cellulose and xylan, but no change for chitin | Functional changes in isolates suggest true evolution through the acquisition of new traits | Isolates may not represent the dominant soil community  Isolates tested under lab conditions (23–25°C) rather than field temperatures (control: 9–11°C; warmed: 14–16°C)  Lab conditions lack ecological interactions |  |
|  |  | Metagenomics | Higher number of carbohydrate-degrading genes in the mineral horizon (0–10 cm), but fewer in the organic horizon (surface soil) | Potential functions are valuable because they represent capacities that could be expressed across a range of conditions instead of one or a few conditions only | Horizons were less clearly separated in the heated plots  Lacks metatranscriptomic analysis to link potential to realized functions |  |
| [Pold et al., 2017](https://paperpile.com/c/NeLSSp/Obgf) | Same as DeAngelis 2015 | Enzyme assays at multiple temperatures | No change in Q10 (no change in slope of biomass-specific enzyme activity, which means no change in enzyme activation energy) | Supports our assumption of fixed enzyme activity Q10 | Lab assays measure potential (not field) enzyme activity | + |
|  |  |  | Higher enzyme production (higher biomass-specific enzyme activity) when accounting for increased temperature in heated plots. At 25°C, Vmax is higher in heated plots for 2 out of 7 enzymes at the first timepoint in mineral soil (and for 1 enzyme in autumn in organic soil), and lower for 1 enzyme. |  | Intercept = [E]*A = enzyme concentration* kinetic activity, with [E] = enzyme allocation*enzyme quantity produced per unit of resource - enzyme turnover. Higher intercept reflects higher enzyme allocation, higher production efficiency, lower turnover, higher kinetic capacity, or all of the above |  |
| [Chen et al., 2018](https://paperpile.com/c/NeLSSp/pQZk) | Meta-analysis (56 experiments, 0.5–23 years, +0.25–5°C) | Enzyme assays at single temperature | Out of the 35 ligninase and 18 cellulase studies with biomass-specific enzyme activity, ligninase activity increased by 40.6% with warming, while cellulase activity showed no consistent response | Enzyme assays better approximate decay potential than respiration measurements | Lacks multi-temperature assays to separate increase in enzyme temperature sensitivity (kinetics) vs. increased production (adaptation)  Lab assays measure potential (not field) enzyme activity | ++ |
|  |  |  | Gradual increase in ligninase activity with warming duration suggests adaptation |  | Requires genomic validation |  |
| [Guo et al., 2020](https://paperpile.com/c/NeLSSp/7XBs) | Temperate grassland (+3°C, 7 years, sandy soil) | Metagenomics | Upregulation (58–80%) of biogeochemical cycling genes (lignin, cellulose, hemicellulose, starch, chitin degradation) | Potential functions are valuable because they represent capacities that could be expressed across a range of conditions instead of one or a few conditions only | Lacks metatranscriptomic analysis to link potential to realized functions | +++ |
|  |  | Heterotrophic respiration (natural temperature fluctuations) | Lower respiration Q10 | Could contradict our assumption of fixed enzyme activity Q10 | Lacks enzyme assays to confirm that the decrease in respiration Q10 is driven by a decrease in enzyme activity Q10 |  |
| [Meng et al., 2020](https://paperpile.com/c/NeLSSp/jJF5) | Meta-analysis (78 experiments, 1–22 years, +0.2–5.7°C) | Enzyme assays at single temperature | Out of 50–80 hydrolytic and 124 oxidative enzyme data points, warming significantly increased oxidative enzyme activity by 9.4% on average, while hydrolytic enzyme responses were inconsistent | Enzyme assays better approximate decay potential than respiration measurements | Lacks biomass-specific normalization to distinguish shift in biomass or per-cell enzyme activity  Lacks assays at multiple temperatures to conclude on kinetics vs adaptive response | + |
|  |  | Fungal/bacterial biomass | Shift in community composition (higher fungal biomass, lower bacterial biomass) | Shifts in community structure indicates eco-evolutionary adaptation, showing that the response is not solely due to plasticity | Minimal shift  Lacks genomic analysis to link with enzyme activity |  |
| [Roy Chowdhury et al., 2021](https://paperpile.com/c/NeLSSp/CoMr) | Same as DeAngelis 2015, but distinct sampling effort (30 years) | Metatranscriptomics | Higher abundances of transcripts for carbohydrate, fatty acid, and lipid metabolism in heated plots, mainly in the organic horizon, but no significant warming effect in major KEGG and CAZy classes. Weak community shifts suggest plasticity rather than eco-evolutionary adaptation. Microbial adaptation may be only indirectly driven by warming via SOC depletion. | Metatranscriptomics provide a snapshot of microbial enzyme expression, including carbon-degrading enzymes, at the time of sampling.  Even if plastic, such adaptation can be a precursor to eco-evolutionary change, aligning with our model predictions. And while adaptive dynamics typically describe eco-evolutionary adaptation, it could be extended to plastic adaptation. | Lacks combination with metagenomic analysis to confirm that this adaptation is eco-evolutionary rather than plastic | ++ |
|  |  |  |  |  |  |  |
|  |  |  |  |  |  |  |
| [Söllinger et al., 2022, 2024](https://paperpile.com/c/NeLSSp/W0ur+Px1W) | ForHot research site (naturally geothermally warmed, 6 weeks–50 years, +3–9°C) | Metatranscriptomics | Higher expression of genes involved in carbohydrate metabolism. Authors interpret this as a physiological response (ie plastic adaptation rather than eco-evolutionary adaptation). |  | Requires reciprocal transplant or genetic analysis for confirmation | ++ |
| [Domeignoz-Horta et al., 2023](https://paperpile.com/c/NeLSSp/4LYOI) | Same as DeAngelis 2015, but distinct sampling effort (13 and 28 years) | Fungi:bacteria ratio | No change in community structure | Suggests that microbial adaptation, if occurring, may not be driven by community structure | Ratio could be stable despite shifts in fungal/bacterial composition | - - |
|  |  | Enzyme assays at multiple temperatures | No change in Q10 | Enzyme assays at multiple temperatures distinguish between enzyme acclimation and microbial adaptation in enzyme production strategy  Supports our assumption of fixed enzyme activity Q10, but provides only weak evidence for microbial adaptation in enzyme production | Lab assays measure potential (not field) enzyme activity |  |
|  |  |  | Higher biomass-specific enzyme activity in warmed samples (suggesting higher enzyme production) for 2 out of 3 enzymes, but only in 1 out of 2 sites and 1 out of 2 seasons |  | Their argument is that this is due to an indirect effect of warming (through lower SOM quality) rather than a direct effect of warming, but would require omics analysis to validate, and it might still apply to validate our model  Lab assays measure potential (not field) enzyme activity |  |
|  |  | Metagenomics | No change in carbon-degrading genes | Potential functions are valuable because they represent capacities that could be expressed across a range of conditions instead of one or a few conditions only |  |  |
| Fan et al. (in review Note S3) | Latitudinal sampling | Metagenomics | Non-linear increase in lignin-degrading gene abundance with warming | Supports our predicted non-linear increase in enzyme allocation with warming | No trend when temperature effects are isolated using a machine-learning model  Reflects adaptation over millions of years rather than decadal-scale changes | - |
| McCulley and Weber 2013-2014 experiment (unpublished, Note S4) | 2 months lab incubation at +4–18°C | Enzyme assays at multiple temperatures | Only 1 out of 6 enzymes aligned with our predictions | Enzyme assays at multiple temperatures distinguish between enzyme acclimation and microbial adaptation in enzyme production strategy | Experiment duration is too short to validate decadal-scale microbial adaptation  Lacks measurements of enzyme concentration and production or turnover to fully conclude | - |

References

[Allison, S. D., Wallenstein, M. D., & Bradford, M. A. (2010). Soil-carbon response to warming dependent on microbial physiology. *Nature Geoscience*, *3*, 336.](http://paperpile.com/b/NeLSSp/kYR1P)

[Carey, J. C., Tang, J., Templer, P. H., Kroeger, K. D., Crowther, T. W., Burton, A. J., Dukes, J. S., Emmett, B., Frey, S. D., Heskel, M. A., Jiang, L., Machmuller, M. B., Mohan, J., Panetta, A. M., Reich, P. B., Reinsch, S., Wang, X., Allison, S. D., Bamminger, C., … Tietema, A. (2016). Temperature response of soil respiration largely unaltered with experimental warming. *Proceedings of the National Academy of Sciences of the United States of America*, *113*(48), 13797–13802.](http://paperpile.com/b/NeLSSp/AQMV)

[Chen, J., Luo, Y., García-Palacios, P., Cao, J., Dacal, M., Zhou, X., Li, J., Xia, J., Niu, S., Yang, H., Shelton, S., Guo, W., & van Groenigen, K. J. (2018). Differential responses of carbon-degrading enzyme activities to warming: Implications for soil respiration. *Global Change Biology*, *24*(10), 4816–4826.](http://paperpile.com/b/NeLSSp/pQZk)

[DeAngelis, K. M., Pold, G., Topçuoğlu, B. D., van Diepen, L. T. A., Varney, R. M., Blanchard, J. L., Melillo, J., & Frey, S. D. (2015). Long-term forest soil warming alters microbial communities in temperate forest soils. *Frontiers in Microbiology*, *6*, 104.](http://paperpile.com/b/NeLSSp/AKxc)

[Domeignoz-Horta, L. A., Pold, G., Erb, H., Sebag, D., Verrecchia, E., Northen, T., Louie, K., Eloe-Fadrosh, E., Pennacchio, C., Knorr, M. A., Frey, S. D., Melillo, J. M., & DeAngelis, K. M. (2023). Substrate availability and not thermal acclimation controls microbial temperature sensitivity response to long-term warming. *Global Change Biology*, *29*(6), 1574–1590.](http://paperpile.com/b/NeLSSp/4LYOI)

[German, D. P., Marcelo, K. R. B., Stone, M. M., & Allison, S. D. (2012). The Michaelis-Menten kinetics of soil extracellular enzymes in response to temperature: a cross-latitudinal study. *Global Change Biology*, *18*(4), 1468–1479.](http://paperpile.com/b/NeLSSp/Q1hNH)

[Guo, X., Gao, Q., Yuan, M., Wang, G., Zhou, X., Feng, J., Shi, Z., Hale, L., Wu, L., Zhou, A., Tian, R., Liu, F., Wu, B., Chen, L., Jung, C. G., Niu, S., Li, D., Xu, X., Jiang, L., … Zhou, J. (2020). Gene-informed decomposition model predicts lower soil carbon loss due to persistent microbial adaptation to warming. *Nature Communications*, *11*(1), 4897.](http://paperpile.com/b/NeLSSp/7XBs)

[Hagerty, S. B., van Groenigen, K. J., Allison, S. D., Hungate, B. A., Schwartz, E., Koch, G. W., Kolka, R. K., & Dijkstra, P. (2014). Accelerated microbial turnover but constant growth efficiency with warming in soil. *Nature Climate Change*, *4*, 903.](http://paperpile.com/b/NeLSSp/fO2LR)

[McCulley, R. L., Bush, L. P., Carlisle, A. E., Ji, H., & Nelson, J. A. (2014). Warming reduces tall fescue abundance but stimulates toxic alkaloid concentrations in transition zone pastures of the U.S. *Frontiers in Chemistry*, *2*, 88.](http://paperpile.com/b/NeLSSp/zsGMR)

[Meng, C., Tian, D., Zeng, H., Li, Z., Chen, H. Y. H., & Niu, S. (2020). Global meta-analysis on the responses of soil extracellular enzyme activities to warming. *The Science of the Total Environment*, *705*(135992), 135992.](http://paperpile.com/b/NeLSSp/jJF5)

[Pold, G., Billings, A. F., Blanchard, J. L., Burkhardt, D. B., Frey, S. D., Melillo, J. M., Schnabel, J., van Diepen, L. T. A., & DeAngelis, K. M. (2016). Long-term warming alters carbohydrate degradation potential in temperate forest soils. *Applied and Environmental Microbiology*, *82*(22), 6518–6530.](http://paperpile.com/b/NeLSSp/sRMX)

[Pold, G., Grandy, A. S., Melillo, J. M., & DeAngelis, K. M. (2017). Changes in substrate availability drive carbon cycle response to chronic warming. *Soil Biology & Biochemistry*, *110*, 68–78.](http://paperpile.com/b/NeLSSp/Obgf)

[Pold, G., Melillo, J. M., & DeAngelis, K. M. (2015). Two decades of warming increases diversity of a potentially lignolytic bacterial community. *Frontiers in Microbiology*, *6*, 480.](http://paperpile.com/b/NeLSSp/tJEE)

[Roy Chowdhury, P., Golas, S. M., Alteio, L. V., Stevens, J. T. E., Billings, A. F., Blanchard, J. L., Melillo, J. M., & DeAngelis, K. M. (2021). The transcriptional response of soil bacteria to long-term warming and short-term seasonal fluctuations in a terrestrial forest. *Frontiers in Microbiology*, *12*, 666558.](http://paperpile.com/b/NeLSSp/CoMr)

[Sinsabaugh, R. L., Manzoni, S., Moorhead, D. L., & Richter, A. (2013). Carbon use efficiency of microbial communities: stoichiometry, methodology and modelling. *Ecology Letters*, *16*(7), 930–939.](http://paperpile.com/b/NeLSSp/Xd5QP)

[Slaughter, L. C., Weintraub, M. N., & McCulley, R. L. (2015). Seasonal effects stronger than three-year climate manipulation on grassland soil microbial community. *Soil Science Society of America Journal. Soil Science Society of America*, *79*(5), 1352–1365.](http://paperpile.com/b/NeLSSp/XqSx7)

[Söllinger, A., Ahlers, L. S., Dahl, M. B., Sigurðsson, P., Le Noir de Carlan, C., Bhattarai, B., Gall, C., Martin, V. S., Rottensteiner, C., Motleleng, L. L., Breines, E. M., Verbruggen, E., Ostonen, I., Sigurdsson, B. D., Richter, A., & Tveit, A. T. (2024). Microorganisms in subarctic soils are depleted of ribosomes under short-, medium-, and long-term warming. *The ISME Journal*, *18*(1). https://doi.org/](http://paperpile.com/b/NeLSSp/W0ur)[10.1093/ismejo/wrae081](http://dx.doi.org/10.1093/ismejo/wrae081)

[Söllinger, A., Séneca, J., Borg Dahl, M., Motleleng, L. L., Prommer, J., Verbruggen, E., Sigurdsson, B. D., Janssens, I., Peñuelas, J., Urich, T., Richter, A., & Tveit, A. T. (2022). Down-regulation of the bacterial protein biosynthesis machinery in response to weeks, years, and decades of soil warming. *Science Advances*, *8*(12), eabm3230.](http://paperpile.com/b/NeLSSp/Px1W)

[Wieder, W. R., Bonan, G. B., & Allison, S. D. (2013). Global soil carbon projections are improved by modelling microbial processes. *Nature Climate Change*, *3*, 909.](http://paperpile.com/b/NeLSSp/g4sKW)
